# Silver and polystyrene nanoparticles activate oestrogen signalling via cytoplasmic oestrogen receptor

**DOI:** 10.1038/s41598-025-30440-4

**Published:** 2025-12-04

**Authors:** Szymon Adam Lekki-Porębski, Sylwia Męczyńska-Wielgosz, Marcin Kruszewski, Agnieszka Grzelak

**Affiliations:** 1 Centre for Digital Biology and Biomedical Science - Biobank Lodz, Faculty of Biology and Environmental Protection, University of Lodz, 90-236 Lodz, Poland; 2 The Bio-Med-Chem Doctoral School of the University of Lodz and Lodz Institutes of the Polish Academy of Sciences, University of Lodz, 90-237 Lodz, Poland; 3 Centre for Radiobiology and Biological Dosimetry, Institute of Nuclear Chemistry and Technology, 03-195 Warsaw, Poland; 4 Department of Molecular Biology and Translational Research, Institute of Rural Health, 20-950 Lublin, Poland

**Keywords:** Oestrogen receptor, Nanoplastics, Breast cancer, Silver nanoparticles, Polystyrene nanoparticles, Biochemistry, Cancer, Cell biology, Environmental sciences

## Abstract

This study explores the endocrine-disrupting potential of two commonly encountered nanomaterials, nanoplastic and silver nanoparticles (AgNPs). Many environmental pollutants, especially endocrine-disrupting chemicals (EDCs), such as bisphenol, heavy metals, interfere with hormone function, posing a serious risk for public health, e.g. prevalence of breast cancer, whose incidence correlates strongly with EDC exposure. However, the impact on hormonal homeostasis of many environmental contaminants, such as nanoplastic, is still unknown. Nanoplastic is a product of the weathering process of plastic goods, while AgNPs are released to the environment from everyday use items, such as health care products, food-related materials or medical devices. These nanoparticles penetrate biological barriers, accumulate in tissues, and may affect oestrogen signalling. Thus, this study investigated how AgNPs and nanopolystyrene (PSNPs) interact with oestrogen receptor (ESR1) signalling in breast cancer cells. The study revealed that in ESR1-positive (ER+) cells, AgNPs notably enhanced ESR1-mediated cell proliferation and progression through the S-phase of the cell cycle, particularly in oestrogen-deprived conditions. The observed effect was ESR1-dependent and effectively blocked by tamoxifen, revealing a ligand-independent activation mechanism. AgNPs downregulated ESR1 signalling-dependent genes, which are linked to cell cycle and proliferation pathways (e.g., IRS1, NCOR1, MED1). PSNPs showed a similar, but milder effect, stimulating ESR1 activation only in the presence of oestrogen (E2). Simultaneous treatment with AgNPs and PSNPs induced a distinct effect, namely, reduced CITED2 and BDNF expression, which was highly dependent on E2 status. The presence of PSNPs also mitigated AgNPs-induced reduction of BRCA1 expression. This study highlights how nanomaterial-induced ESR1 activation can lead to enhanced epithelial-mesenchymal transition and cell cycle progression, suggesting potential adverse effects of nanomaterials in ER+ cancer proliferation via protein kinase-mediated ESR1 modulation.

**Supplementary Information:**

The online version contains supplementary material available at 10.1038/s41598-025-30440-4.

## Introduction

Disruption of human endocrine homeostasis caused by environmental pollutants presents a significant challenge for public health on a global scale. The impact of environmental pollutants on oestrogen-dependent signalling is one of their best-documented adverse health effects. It is now well established that humans are continuously exposed to a constant level of xenoestrogens, due to their ubiquity in the environment and bioaccumulation in a trophic chain^1^. This results in many disorders, such as fertility problems, abnormal development of male sexual organs, menstrual cycle disturbance, disruption of lipid metabolism, and oestrogen-dependent cancers^2^. Among these, breast cancer plays a dominant role, representing the most common type of cancer among women, with 2.3 million new cases each year^3^. The incidence of breast cancer significantly correlates with exposure to multiple endocrine-disrupting chemicals (EDCs)^4^. Despite the growing awareness of organic and non-organic EDCs, the oestrogenic potential of numerous environmental pollutants remains unknown, among them nanomaterials (NM)^5^.

NM can be introduced into the environment intentionally, such as the use of NM-functionalized products, or unintentionally, for instance, as a contamination of wastewater or a product of weathering of a bulk material. The exponential growth in plastic production, combined with its limited recycling, has led to the accumulation of plastic pollutants in the environment. These plastic products degrade both biotically and abiotically, resulting in the formation of microparticles and nanoparticles of plastic^6^. The size of plastic particles has a significant impact on their behaviour in the environment. Larger particles may constitute a separate microenvironment for different organisms, while smaller ones, especially particles smaller than 100 nm, easily pass through biological barriers and penetrate organisms, including humans^7^.

Plastic nanoparticles present in the environment can enter the human body through various routes, including percutaneous, inhalation, and ingestion. Of these, ingestion is the major route of exposure^8^. The mean concentration of nanoplastic in human blood is 1.6 μg/cm^3^ blood, while the maximum concentration exceeds 12 μg/cm^3^ blood in individual cases^9^. The most frequently identified plastic nanoparticles (NPs) in human blood are made of polyethylene and polystyrene (PS). Although the amount of produced polystyrene is much lower than polyethylene, the practice of PS recycling remains at a relatively low level, even in developed countries. This is due to the high cost of PS recycling, resulting in low profitability for such procedures^10^. As a consequence, this results in an increasing quantity of PS deposited in the environment. Moreover, it has been demonstrated that the degradation rate of PS is the highest among the commonly used plastics^11^.

Intentional exposure to NM primarily arises from the utilization of everyday products that have been functionalized with it, thereby imparting novel and distinctive properties to the objects. An illustrative example is silver NPs (AgNPs)^12^, which are widely used due to their antibacterial properties. AgNPs have been employed in a range of products, such as medical devices and instruments, clothes, or food packaging materials, from which they can be released to the environment and penetrate biological barriers. Once in the human body, AgNPs accumulate in the tissue of the brain, liver, lungs, kidneys or breast^13^. In the breast, AgNPs accumulate in mammary gland tissue, which leads to disruption of its epithelial structure and impaired lactation^14^. Our studies revealed that exposure of male rats to AgNPs resulted in adverse effects on hormonal regulation of male reproductive function, in particular alterations of the sex steroid balance and expression of genes involved in steroidogenesis and steroid metabolism, a decrease in sperm count, etc.^15,16^. However, there is still a big knowledge gap related to the effect of nanoplastic and AgNPs on female sex hormone homeostasis, especially verification of their endocrine-disrupting potential.

Thus, the objective of this study was to investigate whether AgNPs and PSNPs, two NMs frequently detected in the human body, have the potential to interfere with oestrogen-dependent signalling. Furthermore, the work examines potential interactions of these NMs on oestrogen-dependent signalling. Our work addresses a research gap related to the endocrine potential of selected NMs.

## Materials and methods

### Materials

All aqueous solutions were made using deionised water from the Milli-Q Integral system (Millipore, Billerica, Massachusetts, USA). The following reagents were purchased from ThermoFisher Scientific (Waltham, Massachusetts, USA): Maxima First Strand cDNA Synthesis Kit for RT-qPCR, PolarScreen™ ER Alpha Competitor Assay, McCoy’s 5A and MEM medium. Fetal Bovine Serum (FBS) was purchased from Eurx (Gdańsk, Poland). Inorganic substrates (acids, bases, salts, etc.) were purchased from ChemPur (Piekary Śląskie, Poland). Anhydrous ethanol was purchased from POCh S.A. (Gliwice, Poland). DTT (Dithiothreitol), anhydrous DMSO (Dimethyl sulfoxide), SDS (Sodium Dodecyl Sulphate), isopropanol, resazurin, propidium iodide, 17-β-oestradiol (E2), polystyrene nanoparticles (PSNPs) and bovine albumin were purchased from Sigma-Aldrich (Saint Louis, Missouri, USA). The Total mRNA Column Isolation Kit was procured from Syngen (Wrocław, Poland). Oestrogen receptor signalling (SAB Target List) H384 Predesigned 384-well panel and SsoAdvanced Universal SYBR Green Supermix were purchased from Bio-Rad (Hercules, California, USA). Silver nanoparticles were purchased from PlasmaChem (Berlin, Germany).

### Nanoparticles preparation

The AgNPs suspension was prepared on the day of the experiment. Four milligrams of nanoparticles were transferred into a 2-millilitre Eppendorf tube, suspended in 1,600 mm^3^ of deionised water. The suspension was sonicated using an Omni Ruptor 4000 Ultrasonic Homogenizer (Omni International, Kennesaw, Georgia, USA) set to the following parameters: power (120 Js^-1^), time (10 min), and pulse power (50%). Once the AgNPs were dispersed, 200 mm^3^ of 10% bovine albumin (w/v) and 200 mm^3^ of 10 × phosphate-buffered saline (PBS) (Ca^2+^ and Mg^2+^ free) were added (final bovine albumin concentration: 1% (w/v)) to simulate the opsonisation of the nanomaterial with bovine albumin and stabilize the nanodispersion. PSNPs suspension was prepared from the manufacturer’s stock solution (50 mg/cm^3^) in deionised water, by adding 10% of (w/v) bovine albumin and 10 × PBS (Ca^2+^ and Mg^2+^ free) to achieve the nanoparticles concentration 2 mg/cm^3^. The received nanomaterial suspension (2 mg/cm^3^) was used next to prepare final concentrations.

### Dynamic light scattering

The size distribution of NPs was measured by dynamic light scattering (DLS) and Nanoparticle Tracking Analysis (NTA). The DLS system measures multiple angles of light scatter and derives a bulk intensity plot of particle sizes. DLS was performed at 25 °C with a scattering angle of 90◦ on the Zeta-sizer Nano ZS (Malvern, Malvern Hills, UK). Stock solutions were diluted 80 times in full exposure media (MEM, DMEM, McCoys’ 5A) and measured in triplicate with 20 sub-runs.

### Cell lines and cell culture

Two model human breast cancer cell lines were employed in this study, MCF-7 (ATCC® HTB-22, ER+ , PR+ , GPER1+ , HER2-) and SK-BR-3 (ATCC® HTB-30, ER-, PR+ , GPER1+ , HER2+), both purchased from the American Type Culture Collection (ATCC, Manassas, Virginia, USA). The cells were cultured in plastic culture flasks with a surface area of 25 cm^2^ or 75 cm^2^. MCF-7 cells were cultured in MEM medium (Sigma-Aldrich, Saint Louis, Missouri, USA) supplemented with NEAA and L-glutamine in accordance with the ATCC recommendations. Routinely, MCF-7 cells were cultured in the presence of E2 at a final concentration of 5 nmol/dm^3^. Alternatively, when an oestrogen-deprived condition was tested, the cells were cultured in medium without oestrogen. The oestrogen-deprived culture was maintained for up to four weeks to prevent the generation of a subculture that was hypersensitive to oestrogens^17^. SK-BR-3 cells were cultured in McCoy’s 5A medium. All media were supplemented with 10% (v/v) FBS. All cells were cultured at 37 °C in a humidified atmosphere (relative humidity of 95%) containing 5% (v/v) CO₂ in a Forma™ Steri-Cult™ CO₂ Incubator (ThermoFisher Scientific, Waltham, Massachusetts, USA). The cultures were monitored for the presence of Mycoplasma spp. contamination using the MycoProbe® Mycoplasma Detection Kit (R&D Systems, Minneapolis, Minnesota, USA) once per 3 months. An overview of methods and models used in this study is presented in Supplementary Table 4.

### Nanoparticle uptake assay

The cells were seeded in a 6-well plate (Thermo Fisher Scientific, Nunclon ™ Delta) at a concentration of 2 × 10^5^ cells/well in a volume of 3 cm^3^ of culture medium. Twenty-four hours after seeding, the nanoparticle suspension was added to each well to achieve the desired final concentration, and the cells were incubated for 2 h under standard conditions. Subsequently, the culture medium was removed and 0.2 cm^3^ of a 0.25% trypsin solution was added to each well. Once the cells had detached from the surface of the culture vessel, 3 cm^3^ of culture medium was added to neutralize the trypsin. Cells were stained in full-growth medium with 5 μg/dm^3^ propidium iodide solution (Invitrogen) to exclude dead cells. The nanoparticle uptake was measured using FACSymphony™ A1 flow cytometer (Becton Dickinson, Franklin Lakes, New Jersey, USA) at FSC, SSC, and PE channel. A representative sample gating was presented in Supplementary Fig. 5B. A total of 1 × 10^4^ events in the last gate were collected, and results were extracted as a median SSC channel value using Flowing Software 2.5.1. As cell lines varied in basic SSC value, the results of the assay were normalized to the control value for each cell line separately. Non-normalized values of SSC parameters for control samples are presented in Supplementary Fig. 6. The concentration of PSNPs was set to an equivalent value to that of the inorganic nanomaterial (AgNPs). This was due to disparities in the molarity of the nanomaterial suspensions, which were caused by random preparation errors. It was therefore reasonable to proceed with subsequent study steps using mixtures of nanomaterials of uniform mass concentration.

### Viability and proliferation assay

The viability and proliferation assay was performed using a resazurin method. The trypsin-released cells were centrifuged (100 × g, 10 min, 21 °C) and suspended in a full growth medium. The density was adjusted to 2 × 10^4^ cells/cm^3^ for MCF-7 cell line or 2.5 × 10^4^ cells/cm^3^ for SK-BR-3 cell line. The cells were seeded in a volume of 0.1 cm^3^ onto 96-well plates with a flat, black bottom (Thermo Fisher Scientific, Nunclon ™ Delta Surface, Waltham, MA, USA). Twenty-four hours after seeding, an appropriate aliquot of the nanoparticle suspension was added to 0.1 cm^3^ of culture medium to achieve the indicated final concentration (in a range of 0.39–100 μg/cm^3^). After the incubation period, a concentrated resazurin solution (in 1X concentrated HBSS) was added to obtain a final resazurin concentration of 15 μg/cm^3^. A change of intensity of the fluorescence signal over time was measured every 20 min for 120 min, at excitation wavelength λ = 571 nm and emission wavelength λ = 585 nm. The results were calculated as the increase in slope value over time. Results of the proliferation assay were normalized to the control value at the 24-h time point for each of the chosen cell lines.

### Wound healing assay

Cells were seeded in a 6-well plate (Thermo Scientific, Nunclon™ Delta) at a concentration of 5 × 10^5^ cells/well in a volume of 3 cm^3^ of culture medium. When the cell cultures reached 100% confluency, a scratch was made in the cell monolayer. The cells were then washed three times with PBS solution, and 3 cm^3^ of cell culture medium containing the appropriate nanoparticle concentration was added. At the start and the end of the incubation period, photographs were taken under a Nikon (Tokyo, Japan) light microscope at the site of each scratch. The surface areas of the scratch at the start and end of incubation were compared using ImageJ software.

### Cell cycle analysis

The cells were seeded in a 6-well plate (Thermo Fisher Scientific, Nunclon ™ Delta) at a concentration of 2 × 10^5^ cells/well in a volume of 3 cm^3^ of culture medium. Twenty-four hours after seeding, the nanoparticle suspension was added to each well to produce a desired final concentration, and the cells were incubated for a further 24 h under standard conditions. Subsequently, the culture medium was removed and 0.2 cm^3^ of a 0.25% trypsin solution was added to each well. Once all cells had detached, 3 cm^3^ of culture medium was added to neutralize the trypsin. Subsequently, the cells were transferred to 15 cm^3^ Falcon tubes and centrifuged (100 × g, 21 °C, 10 min). The supernatant was removed, and the cell pellet was resuspended by adding 0.15 cm^3^ of PBS solution. Subsequently, 0.1 cm^3^ of the cell suspension was injected beneath the surface of 1 cm^3^ of a 70% aqueous ethanol solution that had been cooled to − 20 °C while vortexing. The cell suspension in ethanol was then centrifuged for 10 min at 300 × g and 4 °C. The supernatant was removed and the pellet was resuspended in chilled PBS, washed and resuspended by gentle pipetting in 75 μg/dm^3^ propidium iodide solution (Invitrogen) with the addition of 50 Kunitz units/cm^3^ of RNase A (Sigma Aldrich) in PBS. The cells were then incubated for 30 min in the dark at 37 °C. Subsequently, the samples were placed on ice, and a low-flow-rate cytometric measurement was taken using an FACSymphony™ A1 flow cytometer (Becton Dickinson, Franklin Lakes, New Jersey, USA) in the PE channel. A representative visualisation of a sample gating is presented in Supplementary Fig. 5A. A total of 5 × 10^3^ events were collected, and results were extracted as the median PE channel value, using Flowing Software 2.5.1.

### Measurement of oestrogen-mimicking properties of nanoparticles

The nanoparticles were evaluated for their capacity to bind ESR1 in vitro using the PolarScreen™ ER Alpha Competitor Assay, in accordance with the manufacturer’s instructions. On the day of the experiment, 2X ER Alpha/Fluormone™ ES2 Green Complex (comprising 4.5 nmol/dm^3^ Fluormone™ ES2 Green and 1 × concentrated ESR1) was prepared. Subsequently, 0.01 cm^3^ of the complex was pipetted into a well of a black 384-well plate for fluorometric measurements. Subsequently, a concentrated sample of nanoparticle suspension (previously diluted in ES2 Screening Buffer) was added to the well. The sample was vigorously mixed, covered to protect the reagents from light, and incubated at room temperature for at least two hours. The fluorescence polarisation was then measured using an EnVision® microplate reader (PerkinElmer, Waltham, MA, USA). Measurements were conducted using an excitation polarising filter with a wavelength of 480 nm and an emission polarising S and P filters with a wavelength of 535 nm. Appropriate controls were employed, including a no-receptor assay minimum (measured as a sample supplemented with 20 μmol/dm^3^ E2) and a maximum control (measured as a sample without any competing ligand). The results after background subtraction were calculated as 1000*mP using the following formulae:

$${\text{Fp}}\, = \,{1}000*\left( {\left( {{\text{S}} - {\text{G}}*{\text{P}}} \right)} \right)/\left( {\left( {{\text{S}}\, + \,{\text{G}}*{\text{P}}} \right)} \right),$$

where: S—fluorescence measured using S filter, P—fluorescence measured using P filter, G—instrument G-factor.

#### qPCR assay

The relative level of mRNA transcripts for selected genes was quantified using a quantitative polymerase chain reaction (RT-qPCR) assay. mRNA was isolated using a commercially available kit (Syngen Blood/Cell RNA Mini Kit, Syngen Biotech, Wrocław, Dolnośląskie, Poland) according to the manufacturer’s instructions. The quality and quantity of the RNA were assessed using a Nanodrop spectrophotometer (ThermoFisher Scientific, Waltham, Massachusetts, USA). This involved measuring the RNA absorption peak at λ = 260 nm and comparing the 230/260 nm and 260/280 nm ratios.

A reverse transcription assay was conducted using the Maxima First Strand cDNA Synthesis Kit for RT-qPCR (ThermoFisher Scientific, Waltham, Massachusetts, USA). The following solutions were added to an Eppendorf tube: 4 mm^3^ of reaction buffer, 2 mm^3^ of reverse transcriptase, 2 μg of total isolated RNA, and finally, RNAse-free water was added to bring the total volume to 2 mm^3^. The sample was incubated for 10 min at 25 °C, 30 min at 60 °C and 5 min at 85 °C. The resulting cDNA was then diluted eight times using DNAse-free water and stored at -80 °C until the day of the experiment.

To conduct an oestrogen receptor gene screening, a primer set derived from oestrogen receptor signalling (SAB Target List) H384 Predesigned 384-well panel was utilized. The primer sequences included in the panel can be accessed on the manufacturer’s website (https://commerce.bio-rad.com/en-pl/prime-pcr-assays/predesigned-plate/sybr-green-estrogen-receptor-signaling-sab-target-list-h384?_gl=1*y9eqpt*_gcl_au*MTYzMjI4ODk1My4xNzQ1NTgyNjcw Bio-Rad, Hercules, California, USA). The RT-qPCR mixture was prepared using a 2 × RT-qPCR reaction mix, DNAse-free water and cDNA sample. A volume of 10 µL of reaction mixture was added to a panel of wells containing lyophilized primers. The RT-qPCR reaction was performed in the following setup: 2 min at 95 °C (hot start), 40 cycles consisting of 5 s at 95 °C (denaturation) and 30 s at 60 °C (annealing, polymerization and fluorescence measurement). A melting curve was generated for each sample in a temperature range between 65 °C and 95 °C (0.5 °C step). The results were expressed as Ct from an automatic regression calculation and standardized using housekeeping genes. The following genes were used as a house-keeping gene control: B2M, GUSB, HPRT1, RPLP0 and TBP. A representative visualization of a melting curve for a specific gene was presented in the Supplementary Tab. 3.

#### Statistical analysis of results

Statistical analysis was performed using Graph Pad Prism 9.3.1 software (San Diego, California, USA). Graph Pad Prism 9.3.1 software was also used to prepare all figures. The normality of distribution was checked using the Shapiro–Wilk test. A variance sphericity was tested using the Spearman test. Non-normally distributed results were tested using the non-parametric Kruskal–Wallis test, accompanied by the post-hoc Dunn test. In case of a lack of sphericity, results were tested using the non-parametric Brown-Forsythe test and a post-hoc test was done with an unpaired t-test with Welch correction. Parametric results were tested using a two-way ANOVA and a post-hoc Tukey test. Statistical hypotheses were verified at α < 0.05 significance level. Parametric results were presented as mean and standard deviation. Non-parametric results were presented as a median and interquartile range.

## Results

**Fig. 1:**
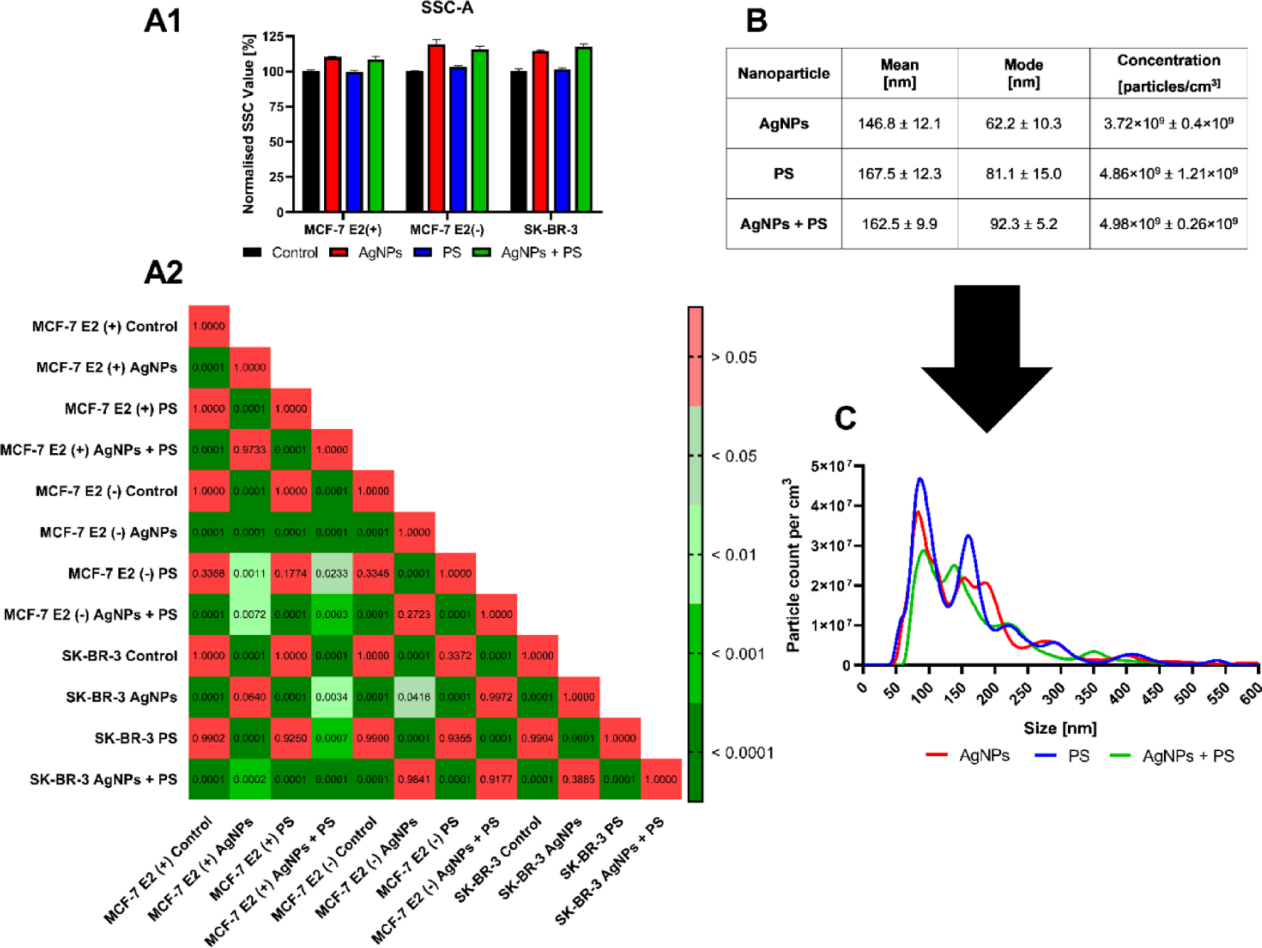
Analysis of properties of chosen NPs and their uptake by cells. Uptake of AgNPs and PSNPs or their mixture after 2 h of incubation (**A1**). *P*-values of post-hoc evaluation of the results are presented in the graph (**A2**). Results are presented as mean ± standard deviation [n = 3]. Characteristics of chosen nanoparticles and their mixture (**B**, **C**). Nanoparticles were suspended in PBS buffer at a final concentration of 10 μg/cm^3^ [n = 3].

The results of the survival assay (see Supplementary Fig. 1A–F and Supplementary Tab. 1A-C) demonstrated that selected nanomaterials exhibited no toxicity to MCF-7 cells. However, AgNPs exert a cytotoxic effect on SK-BR-3 that increased with nanoparticle concentration and duration of incubation period. Subsequently, single concentrations of nanomaterials and mixtures of nanomaterials were selected for further investigation, as outlined in Supplementary Table 2. Due to the negligible toxicity of AgNPs for MCF-7 cells, a concentration of 25 μg/cm^3^ was arbitrarily selected as the main concentration, based on our previous studies^18^. For the SK-BR-3 line, AgNPs concentration corresponding to IC50 value for the 24-h time point was selected. The analysis of nanoparticle uptake (Fig. 1A) demonstrated that AgNPs effectively penetrated cells, irrespective of the presence of nanoplastic. It is noteworthy that the presence of a hormone ligand affected the uptake of the nanomaterial. A comparison of MCF-7 cells, supplemented or not with E2, revealed a statistically significant increase in the uptake of AgNPs under conditions of oestrogen deprivation.

**Fig. 2:**
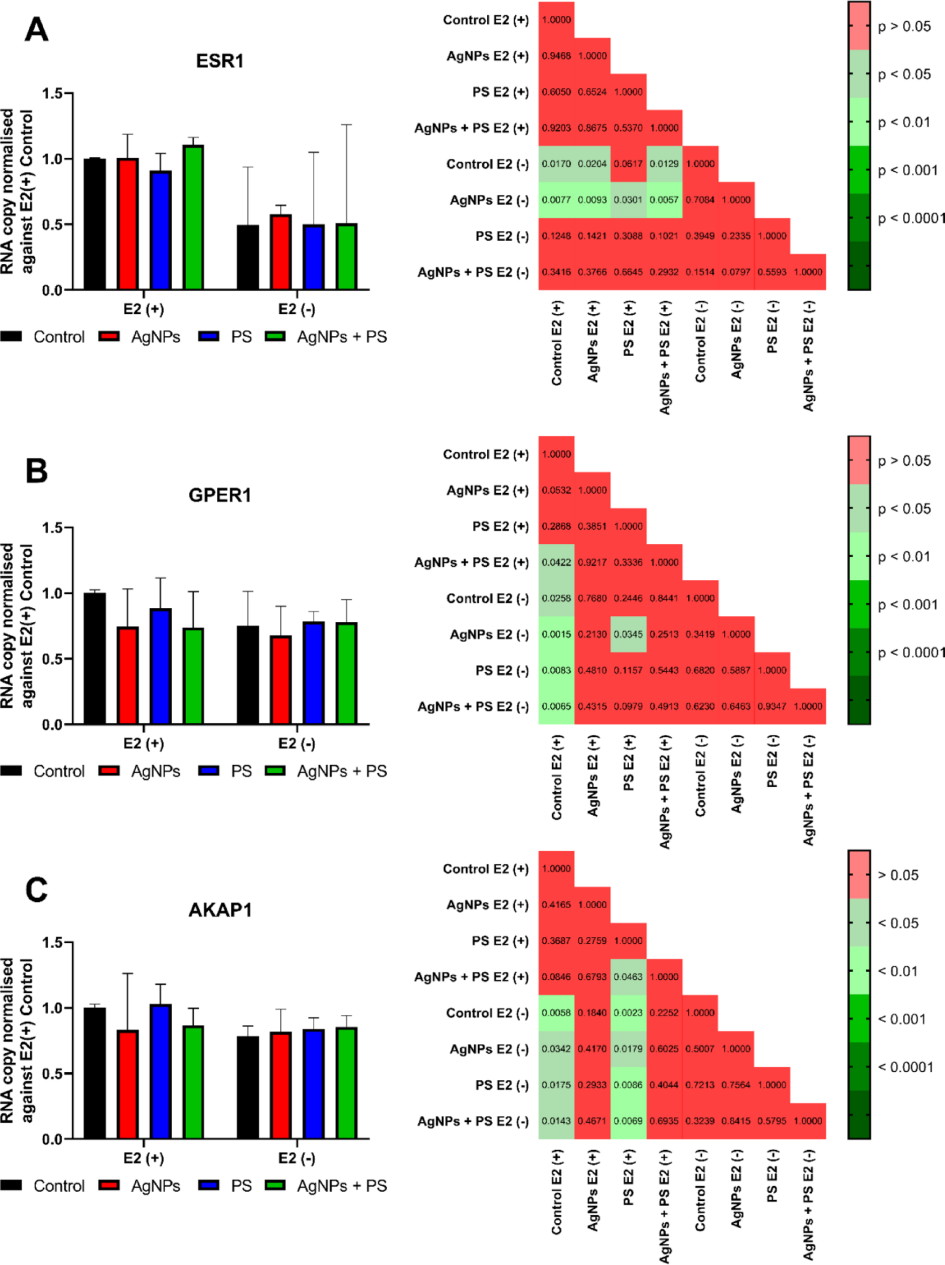
Effects of nanoparticle incubation on transcription of genes coding oestrogen signalling-related proteins in MCF-7 cells. *P*-values of post-hoc evaluation of the results are presented on the right of each graph. Results were tested by non-parametric ANOVA with a post-hoc Tukey test and are presented as a median with interquartile range [n = 7].

**Fig. 3:**
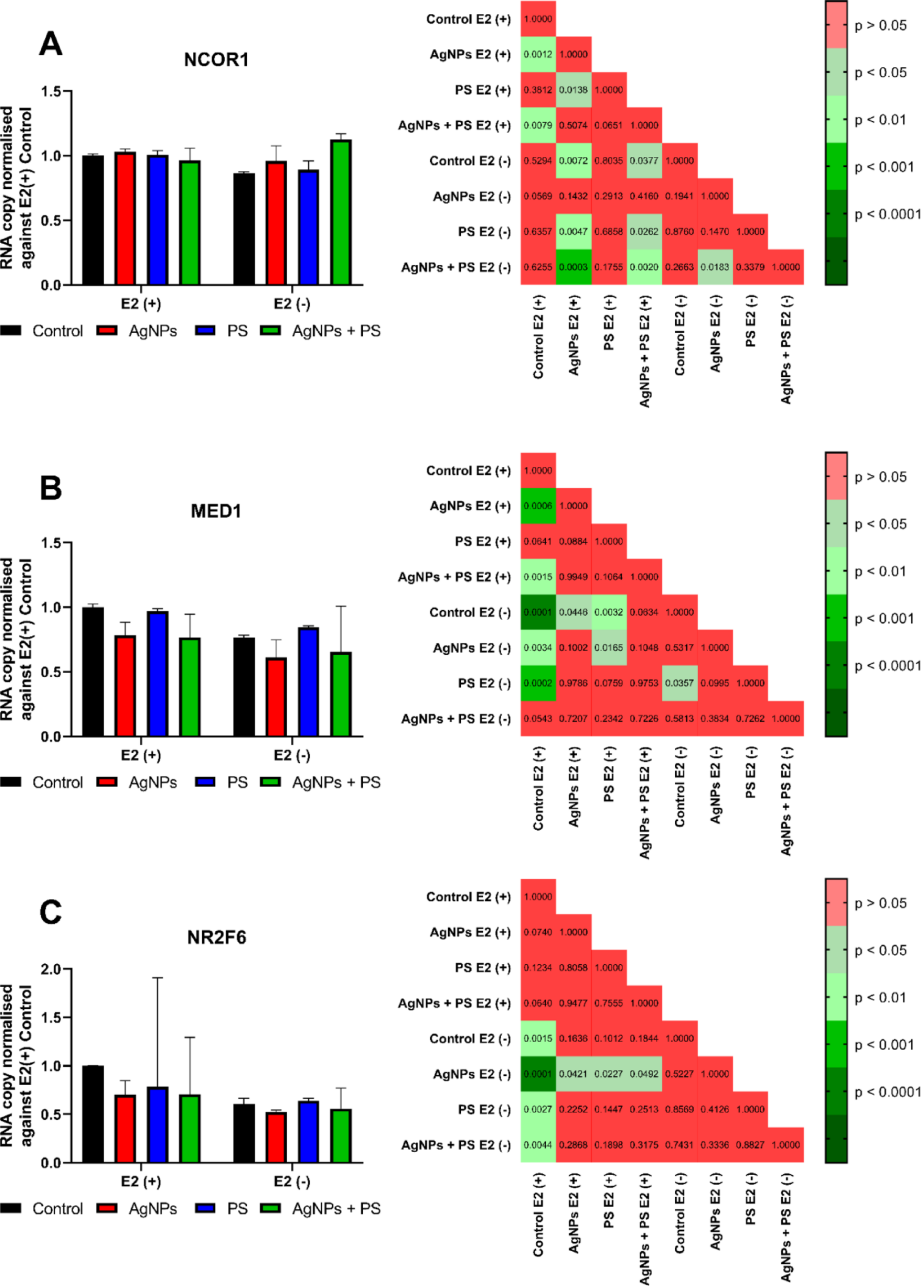
Effects of nanoparticle incubation on transcription of genes coding oestrogen signalling-related proteins in MCF-7 cells and playing a role as nuclear receptor coregulators. *P*-values of post-hoc evaluation of the results are presented on the right of each graph. Results matching requirements for two-way ANOVA (*NCOR1* (**A**)) with a post-hoc Tukey are presented as mean ± standard deviation. Results tested by non-parametric ANOVA (*MED1* (**B**), *NR2F6* (**C**)) with a post-hoc Dunn test are presented as a median with interquartile range [n = 7].

Transcriptional analysis (Fig. 2A–C and Fig. 3A–C) revealed that both NPs modulated the expression of genes associated with oestrogen-dependent signalling pathways. AgNPs treatment, in the presence of E2, significantly reduced transcription of *NCOR1* and *MED1*, whereas no statistically significant alteration was identified in the expression of *NR2F6, AKAP1, GPER1* and *ESR1*. In the absence of E2, AgNPs treatment did not alter statistically significant transcription of any tested genes. PSNPs treatment, in the presence of E2, did not induce alterations in transcription of tested genes; however, in the absence of E2, PSNPs treatment increased *MED1* transcription in comparison to the E2(-) control (Fig. 3B). The mixture of both NPs elicited a unique effect when compared to samples incubated with a single nanomaterial. Treatment with the mixture of NPs reduced transcription of *GPER1, NCOR1* and *MED1* in the E2(+) condition compared to E2(+) control. Moreover, a comparison of the expression of *AKAP1* in the E2(+) culture between samples treated with PSNPs or the NPs mixture reveals a statistically significant difference. Additionally, measurement of *NCOR1* transcription in the E2(-) condition demonstrated a statistically significant difference between AgNPs treatment and the NPs mixture treatment groups. Impact on *NCOR1* transcription was not observed in the E2(+) condition. The collective findings presented in Figs. 2 and 3 suggest the possibility of more intricate interactions between nanomaterials and oestrogen-dependent signalling pathways. This hypothesis is corroborated by the results of in vitro binding studies of nanomaterials to the oestrogen receptor α (ESR1). The results (Supplementary Fig. 3A,B) demonstrated that both AgNPs and PSNPs in the tested concentration did not bind to the LBD domain of the receptor, suggesting that their action was not attributable to a straightforward oestrogen-mimicking mechanism.

**Fig. 4:**
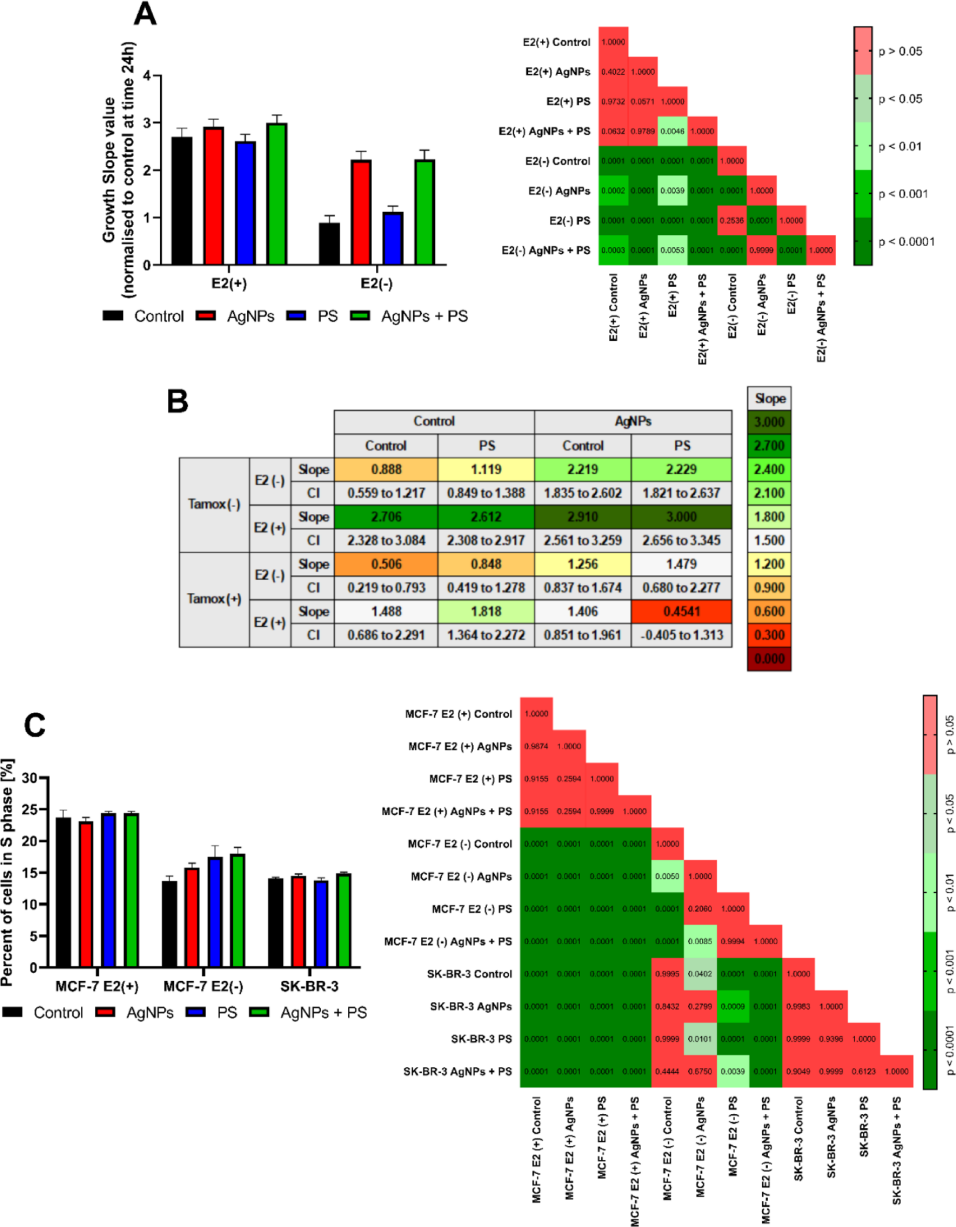
Effects of nanoparticle incubation on (**A**) Proliferation of MCF-7 cells [n = 6], **B** Proliferation rate of cells in comparison to tamoxifen co-incubation [n = 6], **C** Ratio of cells in S phase of the cell cycle [n = 4], *P*-values of post-hoc analysis of results are presented on right side of each graph. Results were tested using two-way ANOVA and are presented as a mean ± standard deviation.

Oestrogens play a pivotal role in the regulation of the proliferation of mammary gland cells, including the management of the passage of cells through G1/S checkpoint. Our results demonstrated that treatment with AgNPs resulted in augmentation of proliferation of MCF-7 cells cultured in oestrogen deprivation conditions (Fig. 4A). The effect was AgNP-specific, as neither treatment with PSNPs alone affected MCF-7 cells’ proliferation, nor simultaneous treatment with PSNPs and AgNPs significantly modified the effect of treatment with AgNPs alone. As illustrated in Fig. 4C, cell cycle analysis revealed that AgNPs treatment increased the number of cells in the S phase of the cell cycle. Interestingly, the increase in the number of cells in S phase was also observed following incubation with PSNPs alone in the E2(-) condition, but apparently did not affect MCF-7 cells’ proliferation. Proliferation of MCF-7 cells was further evaluated following coincubation with a non-toxic concentration of selective oestrogen receptor inhibitor—tamoxifen (3 μmol/dm^3^). Presence of tamoxifen abolished the proliferative effect of AgNPs on MCF-7 E2(-) cells (Fig. 4B), further suggesting that the observed effect was AgNP-specific and ESR1-dependent. For SK-BR-3 cells, none of the incubation variants affected the proliferation rate.

**Fig. 5:**
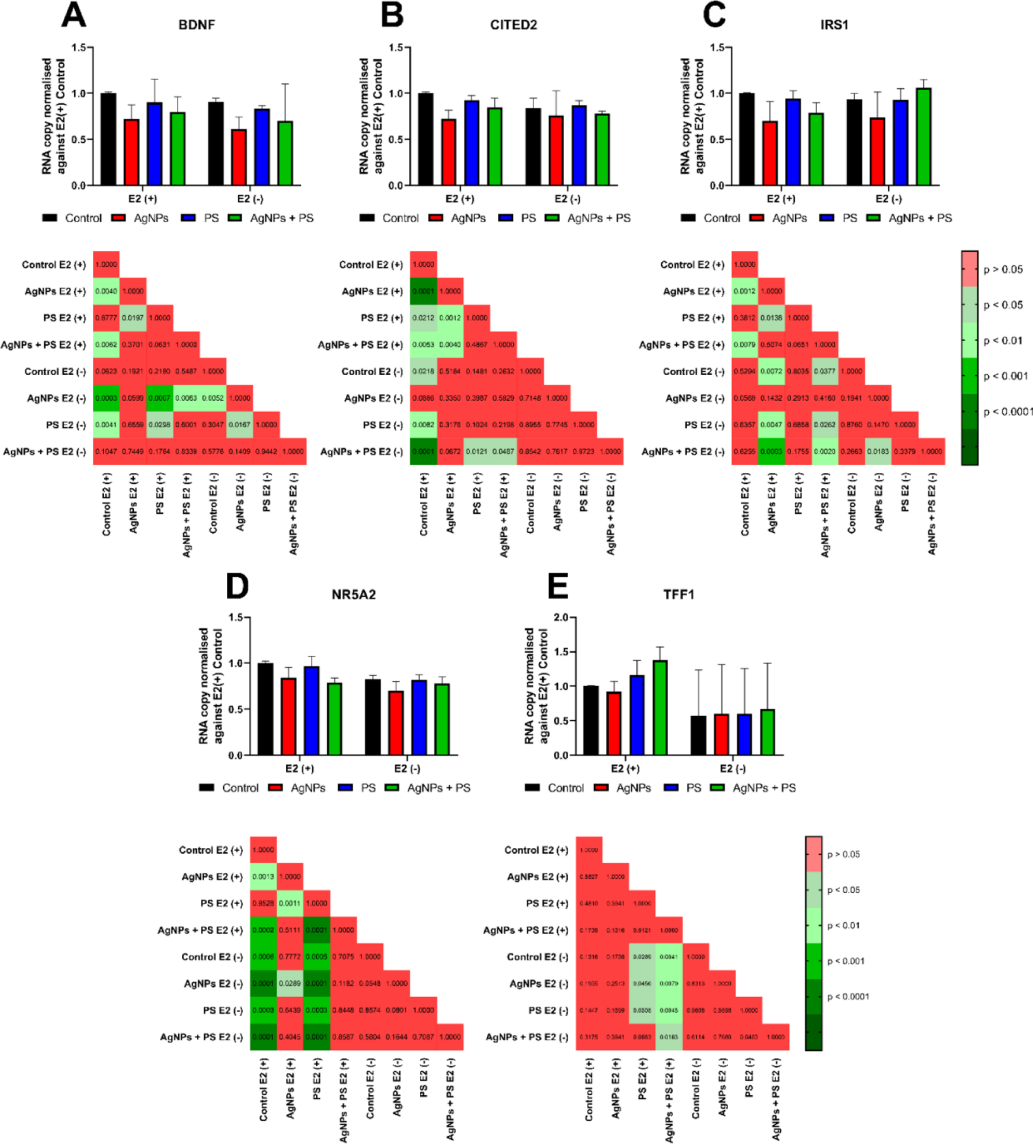
Effects of nanoparticle incubation on transcription of oestrogen-related genes responsible for cell proliferation [n = 7]. *P*-values of post-hoc analysis of results are presented below each graph. Results matching requirements for two-way ANOVA (*IRS1* (**C**), *NR5A2* (**D**)) are presented as a mean ± standard deviation. Results tested by non-parametric ANOVA (*BDNF* (**A**), *CITED2* (**B**), *TFF1* (**E**)) are presented as a median with interquartile range.

Gene expression analysis revealed that the presence of E2 modified the effect of AgNPs or PSNPs treatment on transcription of *TFF1*, *NR5A2* and *IRS1* (Fig. 5C–E). Transcription of *BDNF* was inhibited by AgNPs treatment, irrespective of the presence of E2 (Fig. 5A). Concerning *CITED2*, neither AgNPs nor PSNPs treatments affected the gene expression in the E2(-) condition; however, the presence of E2 resulted in a reduction of *CITED2* transcription, after treatment with AgNPs or PSNPs alone (Fig. 5B). Though combined treatment still diminished *CITED2* transcription, the presence of PSNPs mitigated the impact of AgNPs treatment. In the presence of E2, treatment with AgNPs also resulted in the reduction of *IRS1* and *NR5A2* transcription (Fig. 5C-D, respectively), regardless of the presence of the PSNPs. In contrast, in the absence of E2, PSNPs treatment was able to limit the impact of AgNPs treatment on *IRS1* and *NR5A2* transcription.

**Fig. 6:**
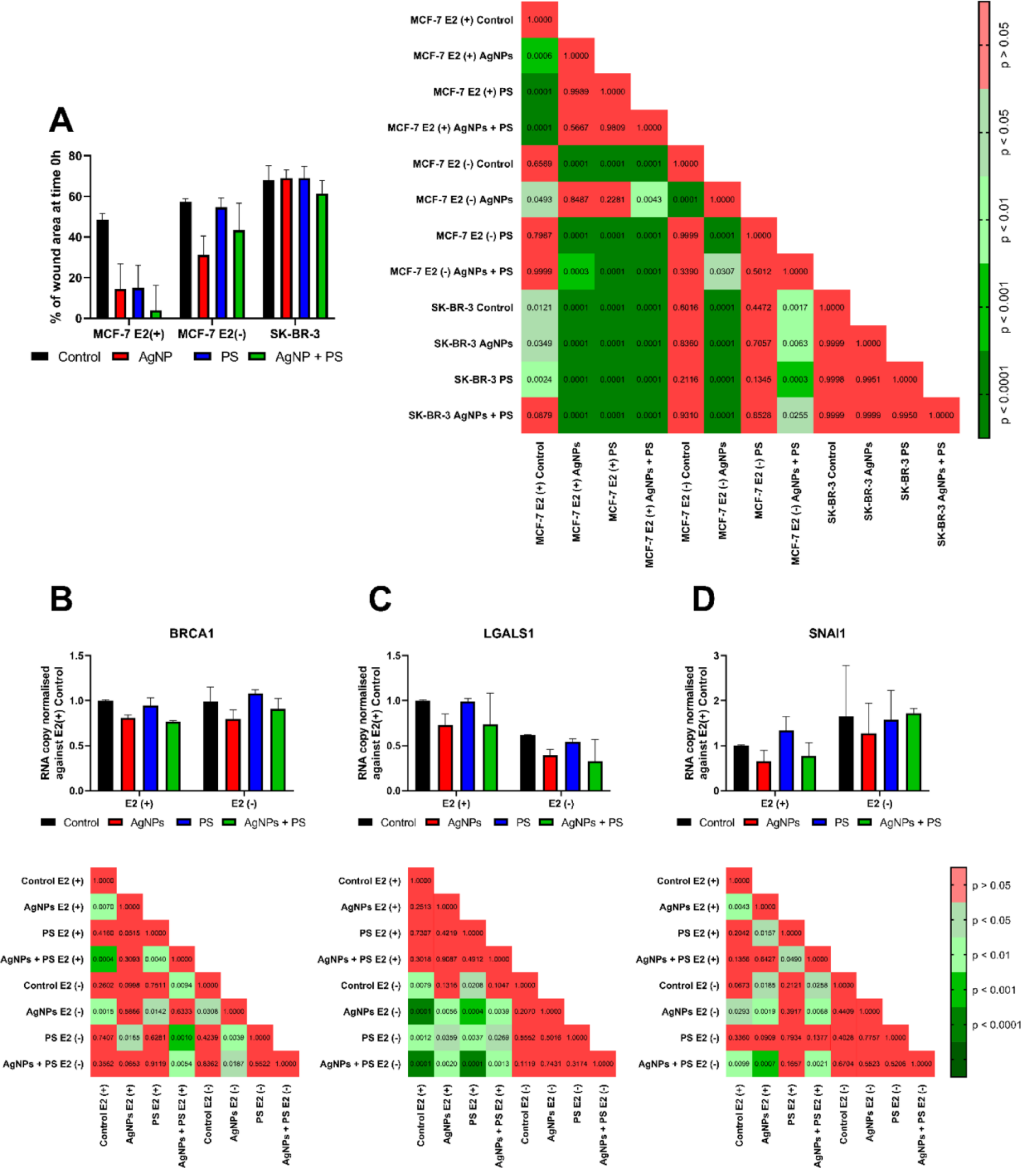
Effects of treatment with NPs on invasiveness parameters of cells. **A** wound healing assay [n = 12] and (**B**–**D**) transcription of oestrogen-related genes responsible for metastatic phenotype [n = 7]. *P*-values of post hoc tests are presented together with every graph. A wound healing assay results (**A**) matched the requirements for two-way ANOVA and are presented as mean ± standard deviation. The other results (*BRCA1* (**B**), *LGALS1* (**C**), *SNAI1* (**D**) were tested by non-parametric ANOVA and are presented as median with interquartile range.

From a clinical perspective, oestrogen-dependent signalling is of pivotal importance in the regulation of epithelial-mesenchymal transition (EMT). Results of the scratch overgrowth assay (Fig. 6A) demonstrated that the status of the oestrogen receptor and its activation by the hormone ligand exerted a significant influence on the effects induced by nanomaterials. No statistically significant changes were observed in SK-BR-3 cells, which did not express the oestrogen receptor. However, in MCF-7 cells, AgNPs treatment sped up the scratch overgrowth, while E2 supplementation further augmented this effect. In cells treated with PSNPs, increased scratch overgrowth was only evident in the presence of oestrogen. The combined treatment revealed a positive, cumulative effect in the presence of oestrogen, while the absence of E2 resulted in a mutual abrogation of the effect of nanomaterials. The analysis of the expression of genes related to the EMT showed that AgNPs treatment, irrespective of the presence of hormonal ligand, caused a decrease in *BRCA1* gene transcription (Fig. 6B), as compared to the appropriate control. PSNPs treatment did not alter transcription of the *BRCA1* gene; however, in the absence of the E2, added PSNPs mitigated the decrease of *BRCA1* expression induced by AgNPs treatment. In the presence of the hormone ligand, the addition of PSPNs did not alter the ability of AgNPs to decrease *BRCA1* expression. Similarly, AgNPs treatment in the presence of E2 decreased transcription of the *SNAI1* gene; however, simultaneous treatment with AgNPs and PSNPs attenuated this effect (Fig. 6D). Interestingly, in the E2-deprivation conditions, AgNPs treatment increased transcription of the *SNAI1* gene. Concerning the *LGALS1* gene (Fig. 6C), no statistically significant changes were evident in response to the treatment with nanoparticles.

## Discussion

The issue of side effects of exposure to nanomaterials and their non-toxic effects on the human body is now receiving increasing attention. The short-term, non-toxic effects of anthropogenic compounds are particularly relevant in the context of oestrogen-dependent signalling. In this work, we verified the effects of the treatment with AgNPs and PSNPs alone or in combination on two human cell lines differing in expression of oestrogen receptor alpha.

Our results revealed that AgNPs were effectively uptaken by MCF-7 and SK-BR-3 cells (Fig. 1A), although MCF-7 cells in an E2-deprived condition revealed the highest potential to uptake AgNPs. Oestrogens regulate ARPC1B and EVL proteins, which are involved in cytoskeleton remodelling and clathrin-dependent endocytosis^17^. As the E2 downregulates the expression of these proteins^19^, its presence likely mitigates the clathrin-dependent nanoparticle uptake^20^.

Estrogen and estrogen mimetics are particularly studied in the context of the development of estrogen-dependent breast cancers. Oestrogen-dependent signalling regulates the transition of the G1/S cell cycle checkpoint of cell cycle and the proliferation of ER+ tumours^21^. Thus, these processes may serve as a marker of estrogen-like activity of the tested compounds^22^. In our hands, AgNPs treatment accelerated proliferation of MCF-7 cells (Fig. 4A) and increased a number of cells in the S phase of the cell cycle (Fig. 4C), however the effect was only evident in E2-unsupplemented cells or in cells supplemented with EF2 but with ESR1 receptor blocked by tamoxifen(Fig. 4B). Furthermore, these effects were not observed in ESR1- and GPER1+ cells (SK-BR-3). These results suggest that AgNPs treatment induces estrogen-dependent proliferation, and a functional ESR1 receptor was essential for the proliferative properties of AgNPs treatment. Moreover, a mechanism of action through the regulation of estrogen receptor transcription was excluded as AgNPs treatment did not lead to changes in *ESR1* or *GPER1* transcription (Fig. 2A and B). One possible explanation for the mechanism of action of AgNPs is the hypothesis of ESR1 receptor activation through a mechanism independent of hormone ligand binding^23^. We hypothesize that AgNPs treatment leads to an increase in the activity of kinases that phosphorylate the oestrogen receptor, such as CK2, AKT1, ERK1/2 or RSK1. These activated kinases would then phosphorylate ESR1, thereby activating it in the absence of a hormonal ligand. This hypothesis is also supported by the lack of AgNPs binding to the receptor in vitro (Supplementary Fig. 3A).

The hypothesis of ligand-independent ESR1 activation by NPs is further supported by the analysis of expression of oestrogen-related genes (Figs. 2, 3 and 5). Moreover, AgNPs treatment reduced transcription of genes, which participate in the regulation of proliferation and apoptosis (i.e. *MED1, NR5A2, CITED2* and *BDNF*). Expression of these genes is either regulated by ESR1-dependent miRNA (i.e. *NR5A2, CITED2* and *BDNF*) or some of them directly or indirectly regulate transcription of ESR1-dependent miRNA (i.e. MED1). The MED1 protein functions as a scaffold protein facilitating the interaction between the ERE-bound ESR1 and the RNA polymerase II complex (RNAPII). In our experiments, the transcription of the *MED1* gene was decreased by AgNPs treatment (Fig. 2E). Although the *MED1* transcript does not correspond strictly to the MED1 protein level, the decrease of *MED1* mRNA caused by AgNPs treatment suggested an impact of AgNPs on the regulation of miRNA transcription. The MED1-ESR1-RNAPII complex has been demonstrated to be essential for the transcription of microRNAs, such as miR-191^33^. The miR-191 is responsible for the decrease of transcripts of *CDK6* and *SATB1* genes and the increase of transcripts of the *BDNF* gene. In our hands, AgNPs treatment resulted in an increase of the *BDNF* gene (Fig. 5A), which confirms involvement of ESR1-dependent miR-191 in effects caused by AgNPs. Another gene regulated by ESR1-dependent miRNA is *NR5A2*. The *NR5A2* gene is responsible for the protection of mammary gland epithelial cells from apoptosis. The *NR5A2* gene is negatively regulated by miR-27b-3p, which is directly induced by activated ESR1^34^. The addition of E2 to AgNPs treatment resulted in a reduction in the transcription of the *NR5A2* gene (Fig. 5D). The last aforementioned gene affected by AgNPs treatment is *CITED2*. The *CITED2* is directly regulated by activated ESR1 through the transcription of miR-145, which results in the repression of *CITED2* expression^35^. The transcription of *CITED2* was decreased in MCF-7 cells following the administration of AgNPs (Fig. 5B). Generally, a reduction in *CITED2* is linked to enhanced migration or proliferation of cancer cells. It is crucial to highlight that the effects of CITED2 downregulation differ depending on tissue type and cross-interaction with other signalling pathways. In some studies, downregulation of the *CITED2* gene, accompanied by downregulation of the *BCL2* gene, led to apoptosis^36^. Since the *BCL2* transcription is upregulated by activated ESR1, the pro-apoptotic effect of *CITED2* downregulation is mitigated^37^. These pathways are governed by ESR1-dependent miRNAs and are independent of miRNAs regulated by GPER1 activity; thus, we concluded that ESR1, rather than GPER1, plays a pivotal role in the oestrogenic effect induced by AgNPs treatment.

The concentrations of AgNPs tested did not bind to the oestrogen receptor in vitro (Supplementary Fig. 3A), which rules out the possibility that AgNPs act as oestrogen mimics. Moreover, AgNPs treatment reduced IRS1 transcription (Fig. 3F). IRS1 forms a complex with ESR1 that interacts with the cytoplasmic end of the receptor for IGF-1 (IGFR), which is responsible for activation of PI3K/AKT (in prepubertal tissue) or ERK1/2 (in post-pubertal tissues) pathways^28^. Activation of ERK1/2 results in the cell cycle arrest at G2/M phase driven by BRCA1^24^ ^30^. Hence, reduction of *IRS1* expression results in a reduction in the number of IRS1/ESR1/IGF-1R complexes, which in turn results in a diminished potential of ERK1/2 to inhibit cell proliferation. As our results demonstrated, the *BRCA1* gene transcription was also reduced by AgNPs treatment (Fig. 6B), we concluded that the PI3K/AKT pathway plays a pivotal role in activation of ESR1-dependent signalling by AgNPs.

In addition to stimulation of cellular proliferation, oestrogens in breast cancer are responsible for the regulation of EMT. Treatment of MCF-7 cells with AgNPs resulted in faster overgrowth of the scratch (Fig. 6A) in the wound healing assay and downregulation of EMT-related genes (Fig. 6B-D). The expression of ESR1 was a critical factor in the observed effect, as it was not observed in ER-negative cells (SK-BR-3). The faster overgrowth of scratch by AgNPs-treated MCF-7 cells might be explained by kinase-driven activation of ESR1 signalling and transcriptional regulation of EMT-associated genes. This hypothesis is confirmed by the effect of AgNPs treatment on *BRCA1* (Fig. 6B), *SNAI1* (Fig. 6D) and *NCOR1* (Fig. 3A) transcription. Treatment with AgNPs reduced transcription of *BRCA1* (Fig. 6B). Repression of *SNAI2* transcription by BRCA1 is a critical point in the regulation of EMT in breast cancer cells^38^. Inhibition of BRCA1 led to increased transcription of the *SNAI2* gene, therefore deregulating processes responsible for EMT control. Furthermore, AgNPs treatment has been observed to result in a reduction in *SNAI1* transcription (Fig. 6D). SNAI1 is known to induce the expression of genes that confer epithelial characteristics to the cell. The reduction in transcription of the *NCOR1* gene was observed under the influence of AgNPs treatment (Fig. 3A). The product of the *NCOR1* gene forms complexes with a dimerised oestrogen receptor that binds to the sequence regulating the expression of the *SNAI2* gene (also known as Slug). In this context, the complex functions as a corepressor, thereby attenuating the severity of EMT processes^25^. Furthermore, oestrogen signalling results in a global reduction in NCOR1 protein levels. NCOR1 decrease occurs via E2-mediated upregulation of the ubiquitin ligase SIAH2, which ubiquitinates NCOR1, leading to its proteolytic degradation^26^.

Our assumption that AgNPs interact with the intracellular protein kinases network, which activates ESR1 via a ligand-independent mechanism, is supported by published results of other authors. AgNPs treatment modulates the activity of PI3K/AKT^26–28^, ERK1/2^29,30^, and the effect on ERK1/2 was reported as early as two hours following incubation with AgNPs^31^. Activation of AKT and ERK kinases by AgNPs treatment is likely a consequence of the binding of released silver ions; however, a form of the nanomaterial is a crucial factor, as administration of analogous doses of silver in salt, such as AgNO₃, did not give similar results^30^. The authors suggested that AgNPs dissolved inside the cell and silver ions had direct access to cytoplasmic enzymes, whereas extracellular silver ions (such as those originating from dissociation of AgNO_3_) were effectively pumped out of the cell by transmembrane copper transporters, due to the chemical similarity of silver and copper ions, and had no chance to interact with the enzymes^32^. Furthermore, in vitro studies have demonstrated that ionic silver was able to activate CK2, a kinase phosphorylating ESR1. CK2 is activated by copper ions, but silver ions can bind the same catalytic subunits of CK2 as copper ions^33^. Thus, our results and literature data lead to the conclusion that AgNPs can activate the ESR1 downstream signalling cascade through a kinase-driven mechanism.

Our experiments also indicated that PSNPs treatment was able to disrupt oestrogen-regulated signalling. The final effect depended on E2 supplementation, as some PSNPs’ effects were observed only in the E2(+) conditions. PSNPs exposure in oestrogen deprivation conditions resulted in an increase in *MED1* transcription (Fig. 3B) and a significant elevation in the proportion of cells in the S phase of the cell cycle (Fig. 4C). MED1 plays a key role in maintaining genome integrity by facilitating base excision repair, DNA mismatch repair, and the cell cycle response to DNA damage^34^. Furthermore, MED1 interact directly with BRCA1, which is phosphorylated by MDC1^35,36^. MDC1 is responsible for the regulation of intra-S phase checkpoints by arresting cell cycle progression during S phase^37^. MED1-BRCA1-MDC1 axis explains the increased proportion of cells in the S phase after PSNPs treatment. Only in the E2(+) condition, PSNPs treatment increased scratch overgrowth (Fig. 6A) and decreased *CITED2* transcription (Fig. 5B). Moreover, PSNPs in tested concentrations did not bind in vitro to ESR1 (Supplementary Fig. 3A,B). Lack of direct binding of PSNPs to ESR1 excludes explanation based on oestrogen-mimicking mechanism. Thus, we hypothesize that PSNPs affect intracellular kinases, which phosphorylate ESR1 after binding E2. Several intracellular kinases phosphorylate the ESR1-E2 complex, such as AKT, PKA and PAK-1^38^. The current state of knowledge confirms that PSNPs treatment has the potential to interfere with signalling based on both the PI3K/AKT and ERK pathways. It was demonstrated that PSNPs treatment induces alterations in the receptors for the ECM via the PI3K/AKT intracellular pathway^39^. It has been demonstrated clearly that oestrogen receptors affect ECM, via complex mechanisms including a network of intracellular kinases^40^. To summarize, in our opinion, the disruption of oestrogen-dependent signalling by PSNPs is based on the disruption of the kinase network, which phosphorylates the ESR1-E2 complex, accelerating effects caused by active ESR1.

The distinctive attributes of the nanomaterial mixture relative to the individual nanoparticles are a result of their physicochemical properties within the incubation environment. PSNPs have a negative surface charge at physiological pH^41^. Nevertheless, a negative surface charge of PSNPs is observed in the absence of a culture medium. In a culture medium, the zeta potential of PSNPs and thus their surface charge decrease significantly towards zero^42,43^. Conversely, AgNPs consistently exhibit a negative charge, with the zeta potential values consistently below -30 mV even in the presence of a medium^44^. This evidence substantiates the stability of the silver nanoparticle suspension. In the mixture, there was an increase in the mode of the nanoparticles’ size distribution and a decrease in the nanoparticle peak at a diameter of less than 100 nm (Fig. 1B and C). There was no increase in a number of particles per cm^3^ according to the theoretical summation of the number of particles in a suspension of individual nanomaterials. Thus, we conclude that PSNPs and AgNPs in the growth medium environment agglomerate, which affects the biological effects of each NPs type.

The results of the experiments demonstrate that the simultaneous treatment with PSNPs and AgNPs resulted in distinctive effects. We were able to separate them into two groups of effects: either specific to the mixture occurring in the E2(+) condition (I) or modulating the effect of a single nanomaterial occurring in the E2(-) conditions (II). No statistically significant changes were observed in the ER(-) SK-BR-3 line (Fig. 4C). Thus, we conclude that ESR1 play a pivotal role in the cumulative effects of nanomaterial mixture. In the oestrogen supplementation condition (group I), the NPs mixture accelerated scratch overgrowth in comparison to a single nanomaterial (Fig. 6A). Apparently, despite agglomeration of AgNPs and PSNPs, AgNPs were able to induce scratch overgrowth via interaction with ESR1-phosphorylating kinases. As we mentioned before, PSNPs had the potency to induce ESR1-phosphorylating kinases after binding the E2, so the scratch overgrowth accelerated by NPs mixture was likely a result of the cumulative effect of both NPs. In the oestrogen deprivation condition (group II), the addition of PSNPs reduced the effect of AgNPs treatment on transcription of *BRCA1* (Fig. 6B), *BDNF* (Fig. 5A) and mitigated the scratch overgrowth by MCF-7 cells (Fig. 6A). In the E2(-) conditions, PSNPs did not exhibit unique properties. Thus biological effect depended both on the availability of E2 and potency of AgNPs to interact with the kinase network phosphorylating ESR1. Common knowledge is that E2 is a ESR1 ligand, which significantly accelerate both proliferation and metastasis of breast cancer. Thus, it is important to analyse overall effects rather than absolute value of change during comparison of groups with different E2 status. In summary, it is clear that the effects of nanomaterial mixture depend on E2 status and not all effects may be explained by simple addition of single-nanomaterial effects.

The findings of this study confirmed that AgNPs and PSNPs present in the human living environment can be treated as EDCs. Both were able to activate and modulate the ESR1-dependent pathway. The experiments provide evidence to support the hypothesis that this was due to the activation of the oestrogen-dependent signalling through kinases that phosphorylate the ESR1. Furthermore, the results demonstrate that contemporary environmental contamination with nanoplastic is an important parameter leading to modification of the effects of other nanoparticles. However, establishing the specific mechanism of action of AgNPs, or PSNPs, and verifying this at the in vivo level requires further research.

## Supplementary Information

Below is the link to the electronic supplementary material.

Supplementary Material 1

## Data Availability

The datasets generated during and/or analysed during the current study are available after direct contact with the corresponding author.

## References

[1] Gea, M., Toso, A. & Schilirò, T. *Estrogenic Activity of Biological Samples as a Biomarker* (Elsevier B.V, 2020). 10.1016/j.scitotenv.2020.140050. 10.1016/j.scitotenv.2020.140050 32927569

[2] Ahn, C. & Jeung, E. B. Endocrine-disrupting chemicals and disease endpoints. *Multidiscip. Digit. Publ. Inst. (MDPI)*10.3390/ijms24065342 (2023). 10.3390/ijms24065342 PMC10049097 36982431

[3] Sung, H. et al. Global cancer statistics 2020: GLOBOCAN estimates of incidence and mortality worldwide for 36 cancers in 185 countries. *CA Cancer J Clin* **71**(3), 209–249. 10.3322/caac.21660 (2021). 33538338 10.3322/caac.21660

[4] Liu, H. et al. *“Endocrine-Disrupting Chemicals and Breast Cancer: A Meta-Analysis* (Frontiers Media SA, 2023). 10.3389/fonc.2023.1282651. 10.3389/fonc.2023.1282651 PMC10665889 38023188

[5] Saxena, S. K. & Khurana, S. M. P. *NanoBioMedicine* (Springer, Singapore, 2020). 10.1007/978-981-32-9898-9.

[6] Zhang, K. et al. *Understanding Plastic Degradation and Microplastic Formation in the Environment: A Review* (Elsevier Ltd, 2021). 10.1016/j.envpol.2021.116554. 10.1016/j.envpol.2021.116554 33529891

[7] Tsochatzis, E. D. et al. *Microplastics and Nanoplastics: Exposure and Toxicological Effects Require Important Analysis Considerations* (Elsevier Ltd, 2024). 10.1016/j.heliyon.2024.e32261. 10.1016/j.heliyon.2024.e32261 PMC11180319 38882323

[8] Krasucka, P. et al. Digestion of plastics using in vitro human gastrointestinal tract and their potential to adsorb emerging organic pollutants. *Sci. Total Environ.*10.1016/j.scitotenv.2022.157108 (2022). 35779726 10.1016/j.scitotenv.2022.157108

[9] Leslie, H. A. et al. Discovery and quantification of plastic particle pollution in human blood. *Environ. Int.*10.1016/j.envint.2022.107199 (2022). 35367073 10.1016/j.envint.2022.107199

[10] Xu, Z. et al. *Progress and Challenges in Polystyrene Recycling and Upcycling* (John Wiley and Sons Inc, 2024). 10.1002/cssc.202400474. 10.1002/cssc.202400474 38757556

[11] Biber, N. F. A., Foggo, A. & Thompson, R. C. Characterising the deterioration of different plastics in air and seawater. *Mar Pollut Bull* **141**, 595–602. 10.1016/j.marpolbul.2019.02.068 (2019). 30955772 10.1016/j.marpolbul.2019.02.068

[12] Nie, P., Zhao, Y. & Xu, H. *“Synthesis, Applications, Toxicity and Toxicity Mechanisms of Silver Nanoparticles: A Review* (Academic Press, 2023). 10.1016/j.ecoenv.2023.114636. 10.1016/j.ecoenv.2023.114636 36806822

[13] Park, E. J. et al. Repeated-Dose Toxicity and Inflammatory Responses in Mice by Oral Administration of Silver Nanoparticles. *Environ. Toxicol. Pharmacol.* **30**(2), 162–168. 10.1016/j.etap.2010.05.004 (2010). 21787647 10.1016/j.etap.2010.05.004

[14] Wang, Z. et al. Effects of silver nanoparticles on maternal mammary glands and offspring development under lactation exposure. *Ecotoxicol. Environ. Saf.*10.1016/j.ecoenv.2023.114869 (2023). 37037110 10.1016/j.ecoenv.2023.114869

[15] Gromadzka-Ostrowska, J. et al. Silver nanoparticles effects on epididymal sperm in rats. *Toxicol. Lett.* **214**(3), 251–258. 10.1016/j.toxlet.2012.08.028 (2012). 22982066 10.1016/j.toxlet.2012.08.028

[16] Dziendzikowska, K. et al. Progressive effects of silver nanoparticles on hormonal regulation of reproduction in male rats. *Toxicol. Appl. Pharmacol.* **313**, 35–46. 10.1016/j.taap.2016.10.013 (2016). 27746313 10.1016/j.taap.2016.10.013

[17] Chan, C. M., Martin, L. A., Johnston, S. R., Ali, S. & Dowsett, M. Molecular changes associated with the acquisition of oestrogen hypersensitivity in MCF-7 breast cancer cells on long-term oestrogen deprivation. *J. Steroid Biochem. Mol. Biol.* **81**(4–5), 333–341 (2002). 12361723 10.1016/s0960-0760(02)00074-2

[18] Rakowski, M., Porębski, S. & Grzelak, A. Silver nanoparticles modulate the epithelial-to-mesenchymal transition in estrogen-dependent breast cancer cells in vitro. *Int. J. Mol. Sci.*10.3390/ijms22179203 (2021). 34502112 10.3390/ijms22179203 PMC8431224

[19] Kumar, A. et al. Estrogen and androgen regulate actin-remodeling and endocytosis-related genes during rat spermiation. *Mol. Cell Endocrinol.* **404**, 91–101. 10.1016/j.mce.2014.12.029 (2015). 25637714 10.1016/j.mce.2014.12.029

[20] Wu, M., Guo, H., Liu, L., Liu, Y. & Xie, L. Size-dependent cellular uptake and localization profiles of silver nanoparticles. *Int. J. Nanomed.* **14**, 4247–4259. 10.2147/IJN.S201107 (2019). 10.2147/IJN.S201107 PMC6559762 31239678

[21] Foster, J. S., Henley, D. C., Bukovsky, A., Seth, P. & Wimalasena, J. Multifaceted regulation of cell cycle progression by estrogen: Regulation of Cdk inhibitors and Cdc25A independent of cyclin D1-Cdk4 function. *Mol. Cell Biol.* **21**(3), 794–810. 10.1128/mcb.21.3.794-810.2001 (2001). 11154267 10.1128/MCB.21.3.794-810.2001 PMC86671

[22] Jeffreys, S. A. et al. *Prognostic and Predictive Value of CCND1/Cyclin D1 Amplification in Breast Cancer With a Focus on Postmenopausal Patients: A Systematic Review and Meta-Analysis* (Frontiers Media S.A., 2022). 10.3389/fendo.2022.895729. 10.3389/fendo.2022.895729 PMC9249016 35784572

[23] Yamnik, R. L. & Holz, M. K. mTOR/S6K1 and MAPK/RSK signaling pathways coordinately regulate estrogen receptor α serine 167 phosphorylation. *FEBS Lett.* **584**(1), 124–128. 10.1016/j.febslet.2009.11.041 (2010). 19925796 10.1016/j.febslet.2009.11.041 PMC8117181

[24] Mebratu, Y. & Tesfaigzi, Y. *How ERK1/2 Activation Controls Cell Proliferation and Cell Death is Subcellular Localization the Answer?* (Taylor and Francis Inc, 2009). 10.4161/cc.8.8.8147. 10.4161/cc.8.8.8147 PMC2728430 19282669

[25] Qureshi, R. et al. Estrone, the major postmenopausal estrogen, binds ERa to induce SNAI2, epithelial-to-mesenchymal transition, and ER+ breast cancer metastasis. *Cell Rep.* **41**, 7. 10.1016/j.celrep.2022.111672 (2022). 10.1016/j.celrep.2022.111672 PMC9798480 36384125

[26] Stanya, K. J. & Kao, H. Y. New insights into the functions and regulation of the transcriptional corepressors SMRT and N-CoR. *Cell Div.*10.1186/1747-1028-4-7 (2009). 19383165 10.1186/1747-1028-4-7 PMC2678994

[27] Zhao, X. et al. Silver nanoparticle-induced phosphorylation of histone h3 at serine 10 involves mapk pathways. *Biomolecules*10.3390/biom9020078 (2019). 30813344 10.3390/biom9020078 PMC6406294

[28] Chang, X. et al. Silver nanoparticles induced cytotoxicity in HT22 cells through autophagy and apoptosis via PI3K/AKT/mTOR signaling pathway. *Ecotoxicol. Environ. Saf.*10.1016/j.ecoenv.2020.111696 (2021). 33396027 10.1016/j.ecoenv.2020.111696

[29] Dayem, A. A. et al. Biologically synthesized silver nanoparticles induce neuronal differentiation of SH-SY5Y cells via modulation of reactive oxygen species, phosphatases, and kinase signaling pathways. *Biotechnol. J.* **9**(7), 934–943. 10.1002/biot.201300555 (2014). 24827677 10.1002/biot.201300555

[30] Saafane, A., Durocher, I., Vanharen, M. & Girard, D. Impact of ultra-small silver nanoparticles of 2 nm (AgNP2) on neutrophil biology: AgNP2 alter the actin cytoskeleton and induce karyorrhexis by a mitogen-activated protein kinase-dependent mechanism in vitro and transitorily attract neutrophils in vivo. *Chem. Biol. Interact.* **365**, 110096. 10.1016/J.CBI.2022.110096 (2022). 35963315 10.1016/j.cbi.2022.110096

[31] Qin, Y. et al. Silver nanoparticles increase connexin43-mediated gap junctional intercellular communication in HaCaT cells through activation of reactive oxygen species and mitogen-activated protein kinase signal pathway. *J. Appl. Toxicol.* **38**(4), 564–574. 10.1002/jat.3563 (2018). 29235124 10.1002/jat.3563

[32] Bertinato, J., Cheung, L., Hoque, R. & Plouffe, L. J. Ctr1 transports silver into mammalian cells. *J. Trace Elem. Med Biol.* **24**(3), 178–184. 10.1016/j.jtemb.2010.01.009 (2010). 20569931 10.1016/j.jtemb.2010.01.009

[33] Chojnowski, J. E. et al. Copper Modulates the Catalytic Activity of Protein Kinase CK2. *Front. Mol. Biosci.*10.3389/fmolb.2022.878652 (2022). 35755824 10.3389/fmolb.2022.878652 PMC9224766

[34] Nagpal, N. et al. Essential role of MED1 in the transcriptional regulation of ER-dependent oncogenic miRNAs in breast cancer. *Sci. Rep.*10.1038/s41598-018-29546-9 (2018). 30087366 10.1038/s41598-018-29546-9 PMC6081450

[35] Honjoh, H. et al. MED1, a novel binding partner of BRCA1, regulates homologous recombination and R-loop processing. *Sci. Rep.*10.1038/s41598-022-21495-8 (2022). 36229463 10.1038/s41598-022-21495-8 PMC9561711

[36] Lou, Z., Silva Chini, C. C., Minter-Dykhouse, K. & Chen, J. “Mediator of DNA damage checkpoint protein 1 regulates BRCA1 localization and phosphorylation in DNA damage checkpoint control. *J. Biol. Chem.* **278**(16), 13599–13602. 10.1074/jbc.C300060200 (2003). 12611903 10.1074/jbc.C300060200

[37] Goldberg, M. et al. MDC1 is required for the intra-S-phase DNA damage checkpoint. *Nature* **421**(6926), 952–956. 10.1038/nature01445 (2003). 12607003 10.1038/nature01445

[38] Grinshpun, A., Chen, V., Sandusky, Z. M., Fanning, S. W. & Jeselsohn, R. *ESR1 Activating Mutations: From Structure to Clinical Application* (Elsevier B.V, 2023). 10.1016/j.bbcan.2022.188830. 10.1016/j.bbcan.2022.188830 PMC13423459 36336145

[39] Chang, J. et al. Airborne polystyrene nanoplastics exposure leads to heart failure via ECM-receptor interaction and PI3K/AKT/BCL-2 pathways. *Sci. Total Environ.* **954**, 176469. 10.1016/J.SCITOTENV.2024.176469 (2024). 39317253 10.1016/j.scitotenv.2024.176469

[40] Mangani, S., Piperigkou, Z., Koletsis, N. E., Ioannou, P. & Karamanos, N. K. *Estrogen Receptors and Extracellular Matrix: The Critical Interplay in Cancer Development and Progression* (John Wiley and Sons Inc., 2024). 10.1111/febs.17270. 10.1111/febs.17270 PMC11970714 39285617

[41] Xu, H. & Casabianca, L. B. Probing driving forces for binding between nanoparticles and amino acids by saturation-transfer difference NMR. *Sci. Rep.*10.1038/s41598-020-69185-7 (2020). 32704150 10.1038/s41598-020-69185-7 PMC7378059

[42] Cortés, C. et al. Nanoplastics as a potential environmental health factor: Effects of polystyrene nanoparticles on human intestinal epithelial Caco-2 cells. *Environ. Sci. Nano* **7**(1), 272–285. 10.1039/c9en00523d (2020).

[43] Tavakolpournegari, A. et al. Hazard assessment of different-sized polystyrene nanoplastics in hematopoietic human cell lines. *Chemosphere*10.1016/j.chemosphere.2023.138360 (2023). 36905991 10.1016/j.chemosphere.2023.138360

[44] Lankoff, A. et al. The effect of agglomeration state of silver and titanium dioxide nanoparticles on cellular response of Hep G2, A549 and THP-1 cells. *Toxicol. Lett.* **208**(3), 197–213. 10.1016/j.toxlet.2011.11.006 (2012). 22108609 10.1016/j.toxlet.2011.11.006

