# Supplementary material for: Silver and polystyrene nanoparticles activate oestrogen signalling via cytoplasmic oestrogen receptor
Source: Sci Rep. 2025 Dec 4;16:948. doi: 10.1038/s41598-025-30440-4 (PMC12783835; doi:10.1038/s41598-025-30440-4)
Supplement: Supplementary file 1 — Supplementary Material 1 [file 41598_2025_30440_MOESM1_ESM.doc]

**Supplementary materials**

**Silver and Polystyrene nanoparticles activate oestrogen signalling via cytoplasmic oestrogen receptor**


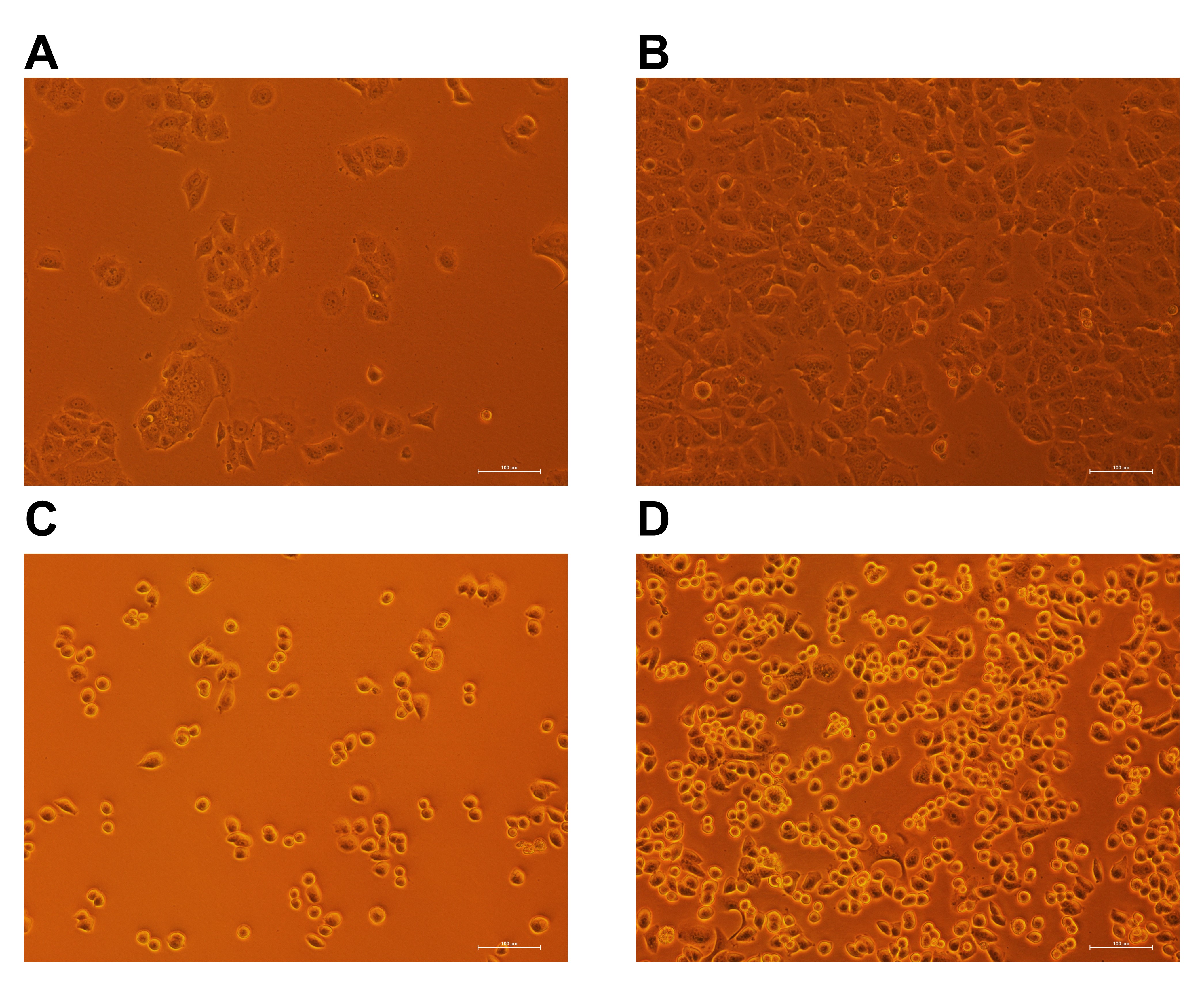


Supplementary Fig. 1A-D: Representative photographs of MCF-7 (A-B) and SK-BR-3 (C-D) cell lines at low density (A,C) and high density (B,D).





Supplementary Fig. 2A-F.: Viability curves of cells incubated 24-72 h with suspension of single nanomaterial. Viability was measured using resazurin assay. Non-linear curves were calculated using GraphPad Prism 8.0 software. Results are presented as mean normalised to untreated control ± SD, n=6.

| **A:** 24 hours | Cell line | | | | | |
| --- | --- | --- | --- | --- | --- | --- |
| Nanoparticle type | MCF-7 E2(+) | | MCF-7 E2(-) | | SK-BR-3 | |
| Parameter | IC_50_ | 95% CI | IC_50_ | 95% CI | IC_50_ | 95% CI |
| AgNPs | - | - | - | - | 54,25 | 42,08 – 85,82 |
| PS | - | - | - | - | - | - |

| **B:** 48 hours | Cell line | | | | | |
| --- | --- | --- | --- | --- | --- | --- |
| Nanoparticle type | MCF-7 E2(+) | | MCF-7 E2(-) | | SK-BR-3 | |
| Parameter | IC_50_ | 95% CI | IC_50_ | 95% CI | IC_50_ | 95% CI |
| AgNPs | - | - | - | - | 38,49 | 34,02 – 43,41 |
| PS | - | - | - | - | - | - |

| **C:** 72 hours | Cell line | | | | | |
| --- | --- | --- | --- | --- | --- | --- |
| Nanoparticle type | MCF-7 E2(+) | | MCF-7 E2(-) | | SK-BR-3 | |
| Parameter | IC_50_ | 95% CI | IC_50_ | 95% CI | IC_50_ | 95% CI |
| AgNPs | - | - | - | - | 24,19 | 21,07 – 27,61 |
| PS | - | - | - | - | - | - |

Supplementary Table 1A-C.: IC_50_ values and 95% confidence intervals of cells incubated 24-72h with suspension of single nanomaterial. Viability was measured using resazurin assay. IC_50_ values and 95% confidence intervals were calculated using GraphPad Prism 8.0 software, n=6.

|  | Cell line | | |
| --- | --- | --- | --- |
| Nanoparticle type | MCF-7 E2(+) | MCF-7 E2(-) | SK-BR-3 |
| AgNPs | 25 μg/cm^3^ | 25 μg/cm^3^ | 54,25 μg/cm^3^ |
| PS | 25 μg/cm^3^ | 25 μg/cm^3^ | 25 μg/cm^3^ |

Supplementary Table 2: Chosen single-point concentrations of nanoparticles used for the rest of experiments in 24-hour incubation variant.


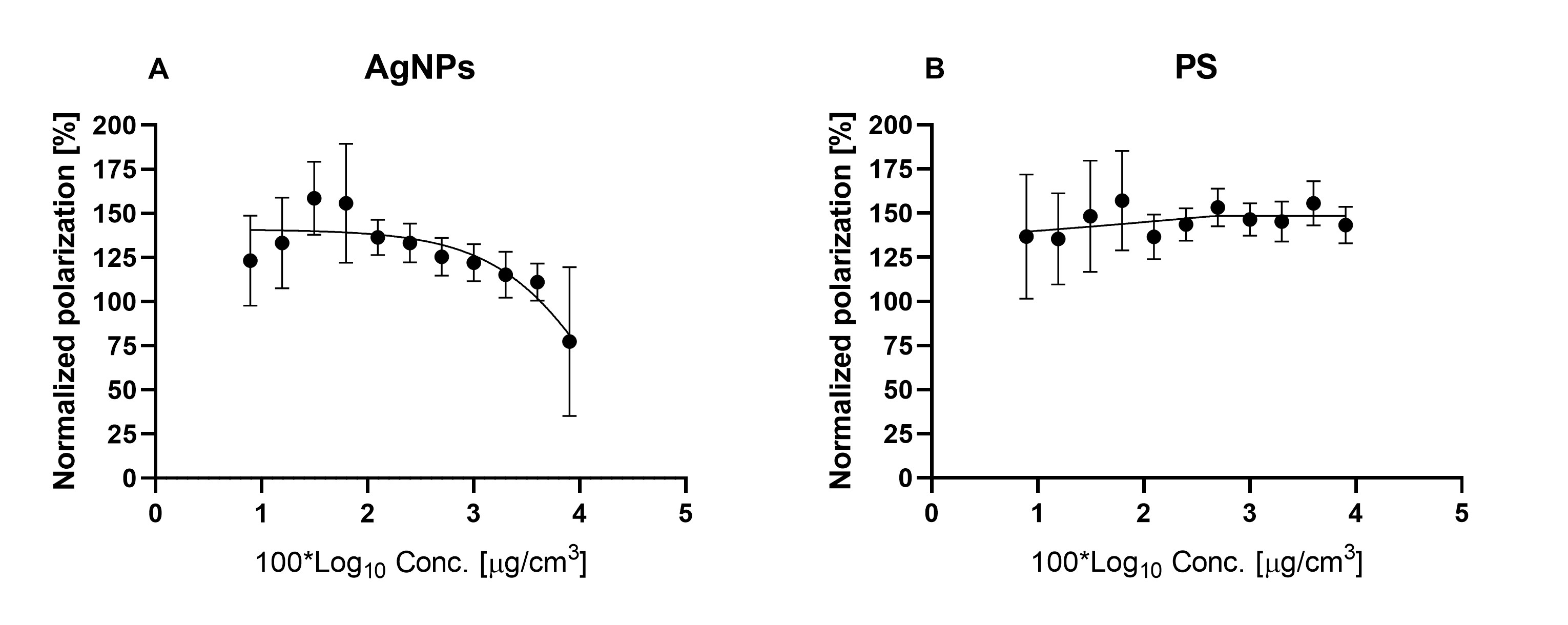


Supplementary Fig. 3: Values of fluorescence polarization obtained with PolarScreen™ ER Alpha Competitor Assay. Results presents biding potential of AgNPs (A) or PS (B) to isolated ERα. Non-linear curves were calculated using GraphPad Prism 8.0 software. Results are presented as mean normalised to untreated control ± SD, n=20.

|  | **A** | | **B** |
| --- | --- | --- | --- |
| Cell discrimination | 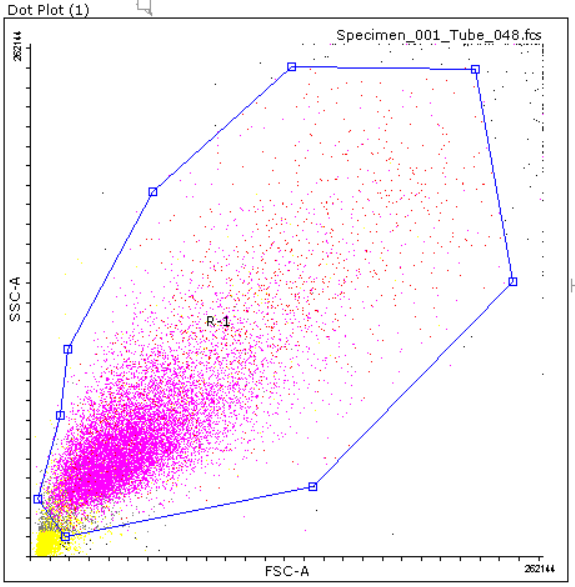 | 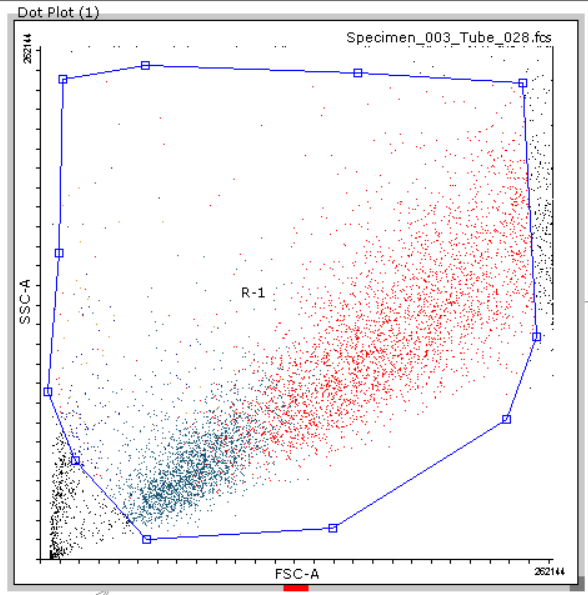 | |
|  |  |  | |
| Singlet cell discrimination (based on size) | 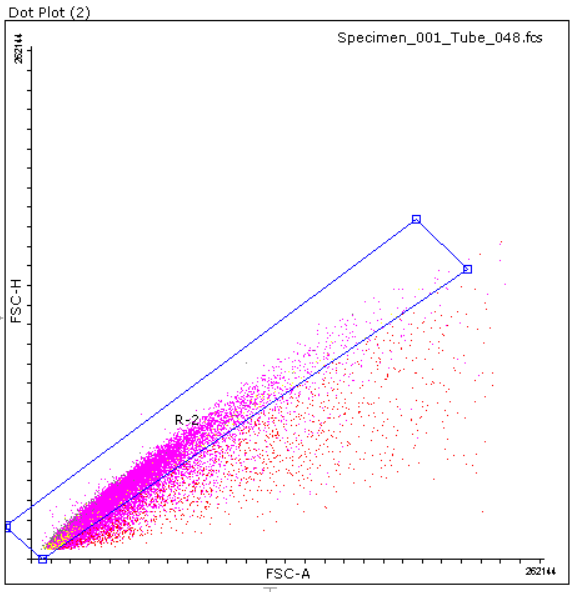 | 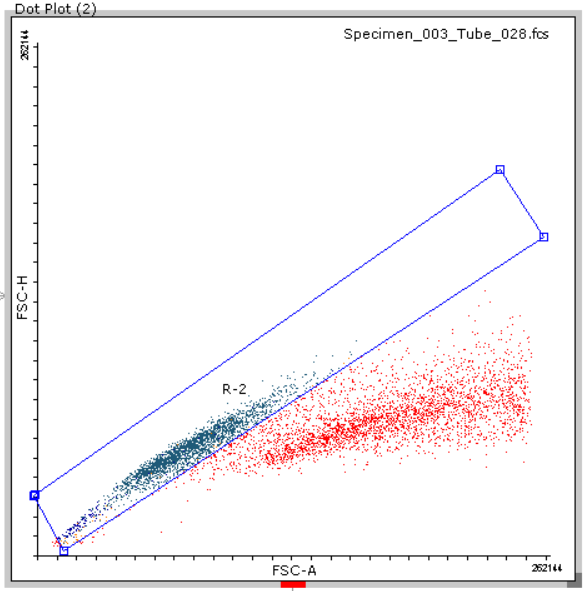 | |
|  |  |  | |
| Singlet cell discrimination (based on granluarity) | 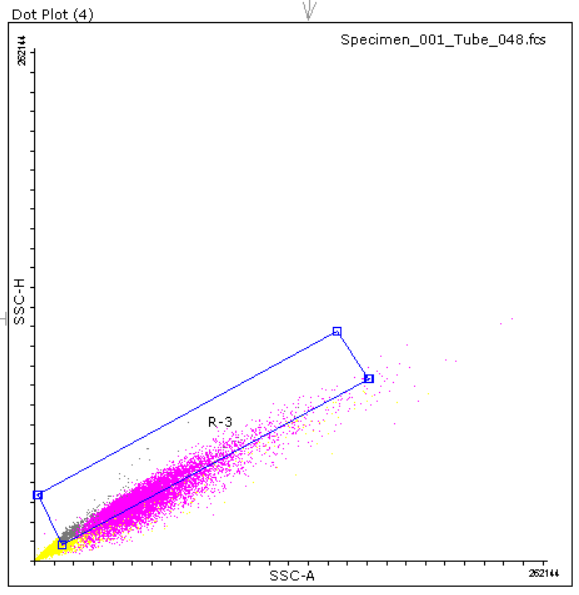 | 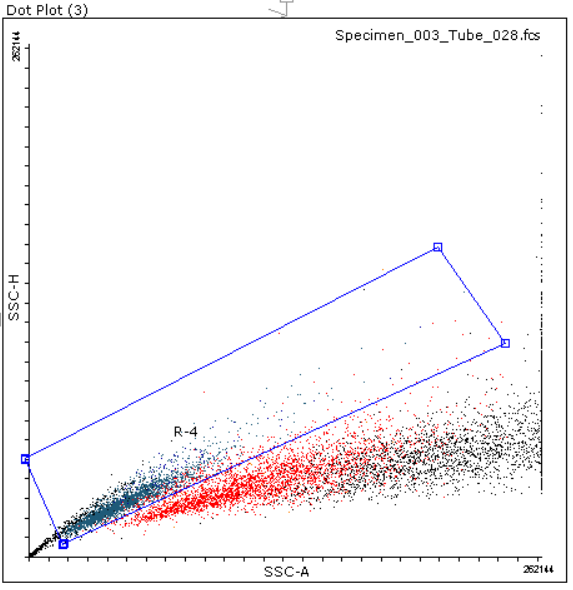 | |
|  |  |  | |
| Live cell discrimination | 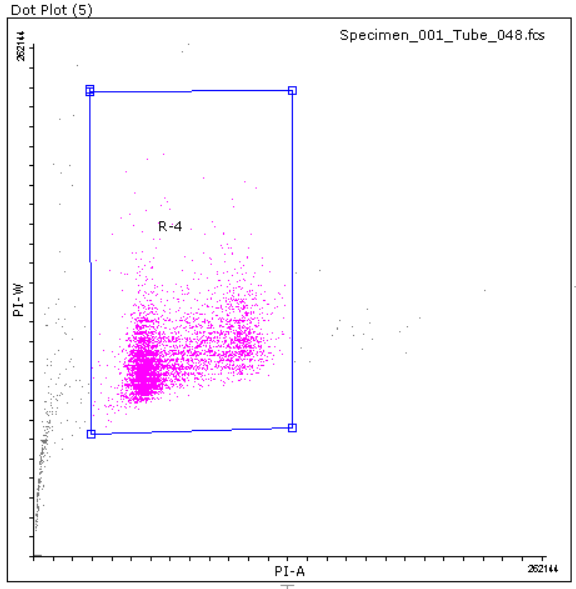 | 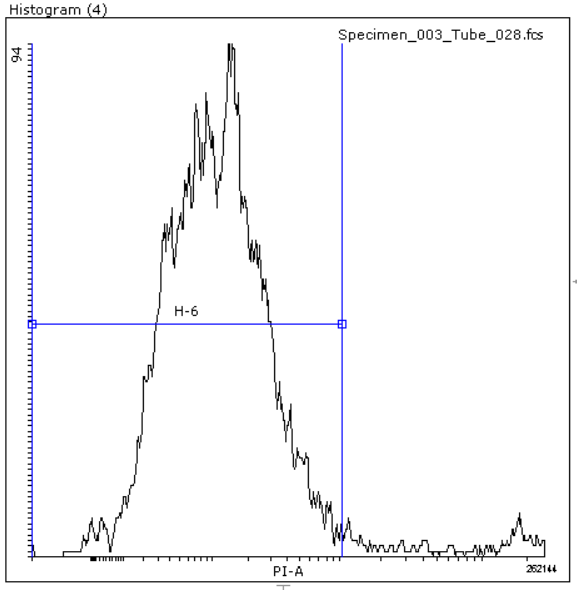 | |
|  |  |  | |
|  | 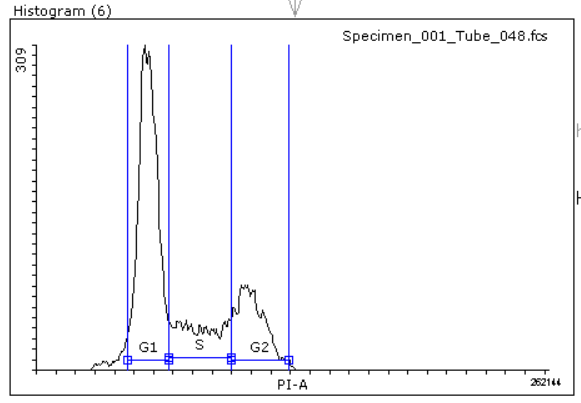 | 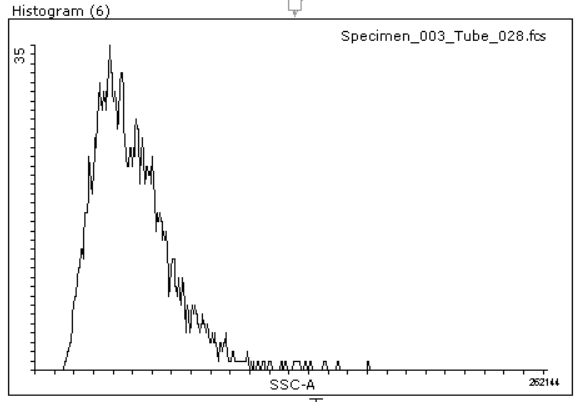 | |

Supplementary Fig. 4: FSC, SSC and PE channel gating strategies used in aim to discriminate artifacts during flow cytometry-based assays. Gating strategies were used respectively in cell cycle (A) and cell loading (B) assays. Gating diagrams were derived from FlowingSoftware 2.0


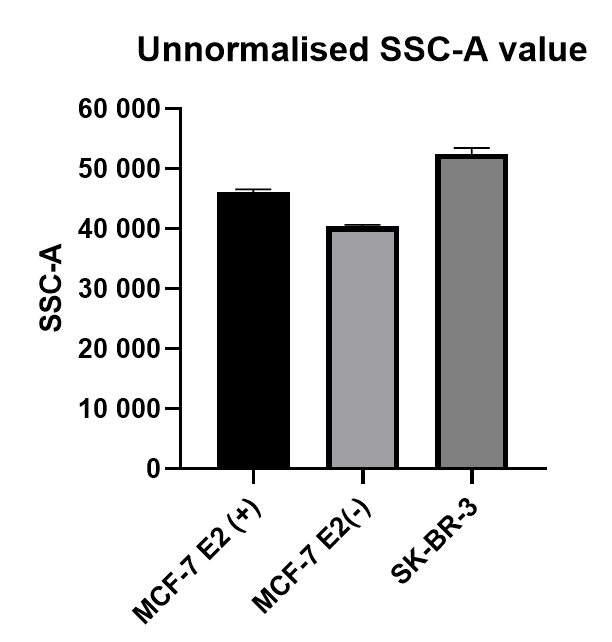


Supplementary Fig. 5: Non-normalised values of SSC parameter achieved using cell loading assay. Results were calculated using GraphPad Prism 8.0 software and presented as mean normalised to untreated control ± SD, n=3.





Supplementary Fig. 6: Heatmap of expression of oestrogen-dependent genes in MCF-7 cell line. Each results was normalised to E2(+) control from each experiment, where value of E2(+) control = 1.0. Heatmap was prepared using GraphPad Prism 8.0 software. Results are presented as median value derived from all experimental values, n = 6.

| BCL2L1 | EBAG9 | PTCH1 | TFF1 |
| --- | --- | --- | --- |
| 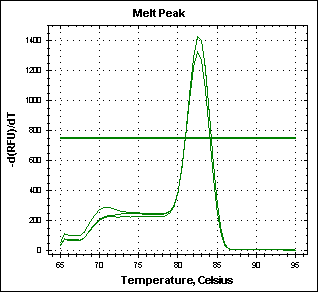 | 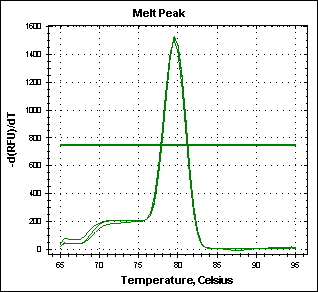 | 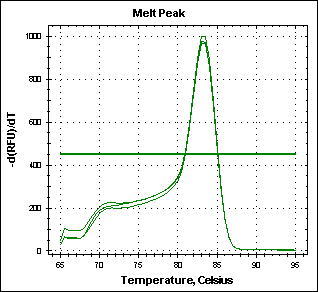 | 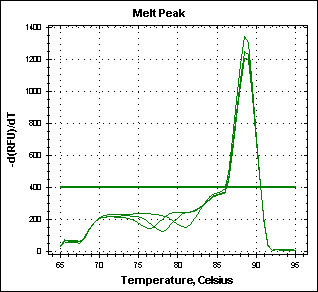 |
| WNT4 | WNT5A | BRCA1 | EFNA5 |
| 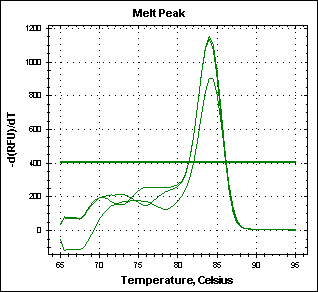 | 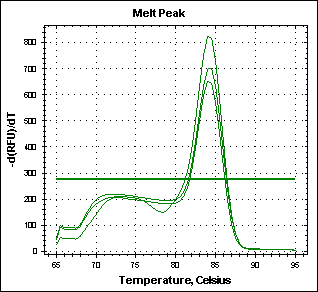 | 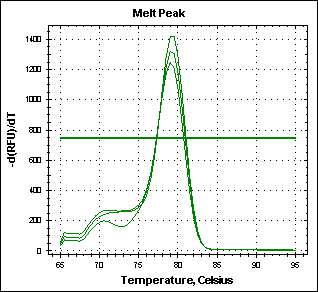 | 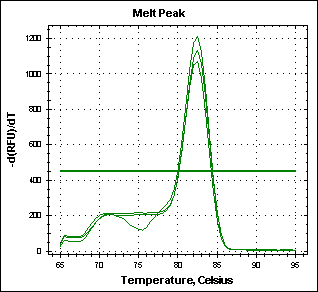 |
| L1CAM | LGALS1 | MMP9 | MTA1 |
| 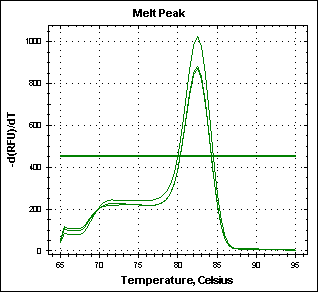 | 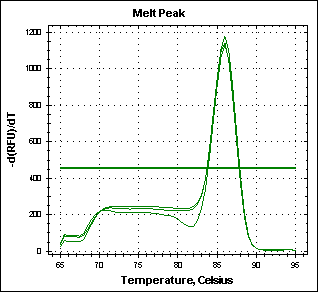 | 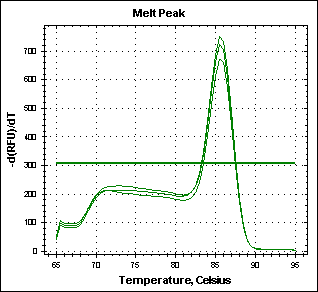 | 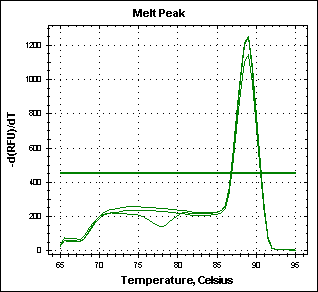 |
| SNAI1 | TGFB3 | THBS1 | WISP2 |
| 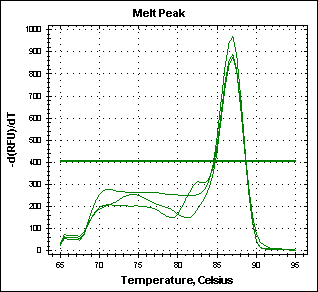 | 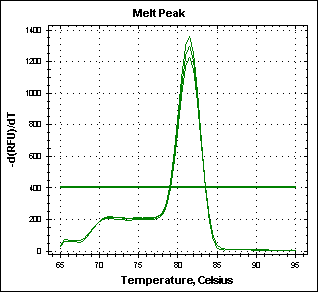 | 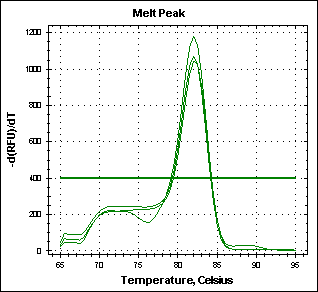 | 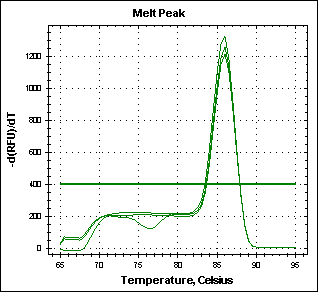 |
| AHR | AKAP1 | ESR1 | FOXA1 |
| 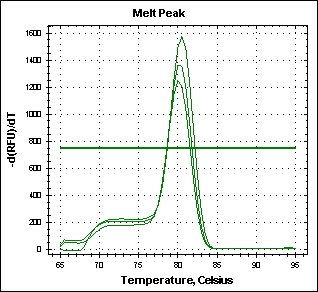 | 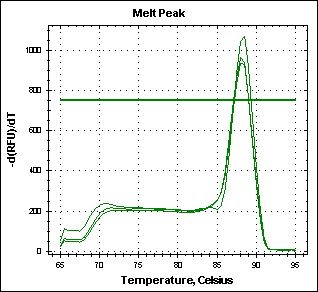 | 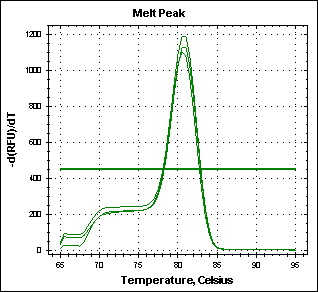 | 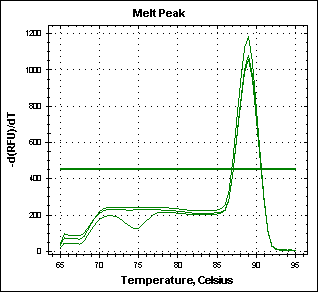 |
| GPER | IGFBP5 | IRS1 | MED1 |
| 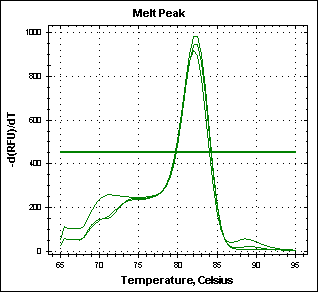 | 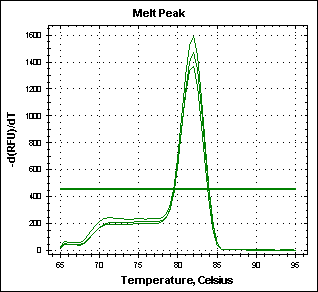 | 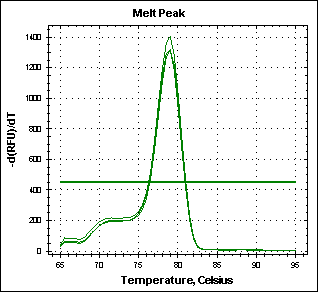 | 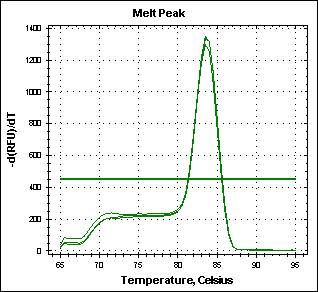 |
| NCOA2 | NCOR1 | SAFB | NR5A2 |
| 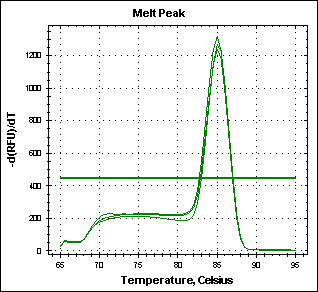 | 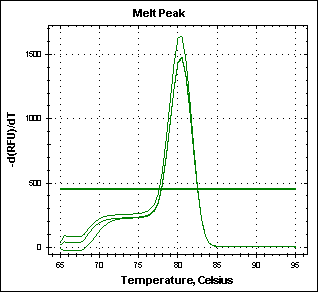 | 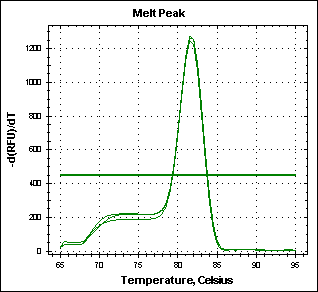 | 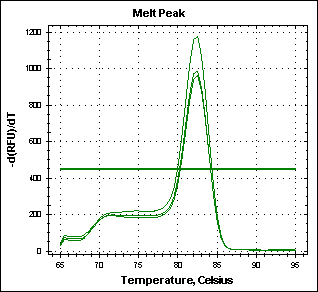 |
| TGFA | BDNF | CITED2 | CKB |
| 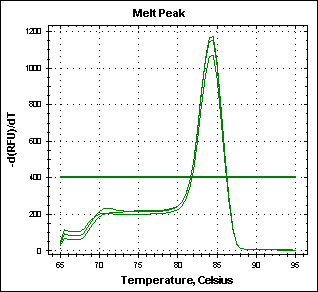 | 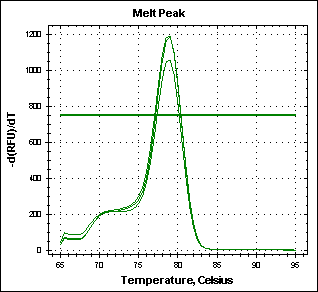 | 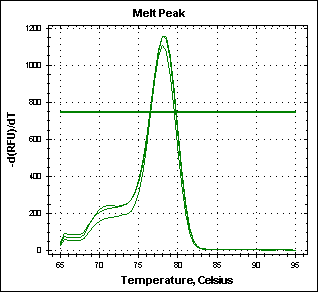 | 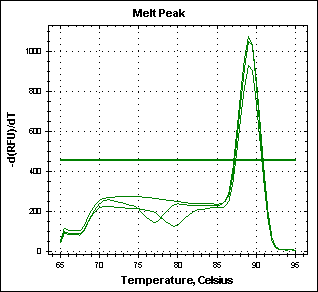 |
| ERBB3 | FST | JUNB | LPL |
| 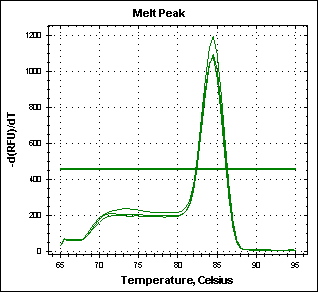 | 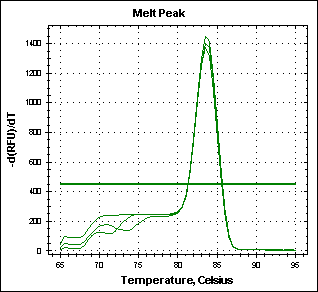 | 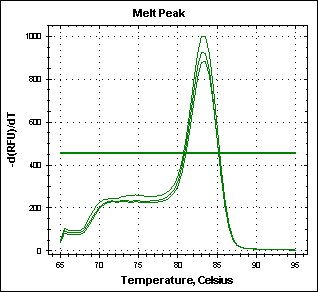 | 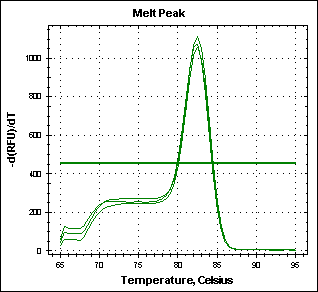 |
| LTBP1 | MAFF | MYC | NR2F6 |
| 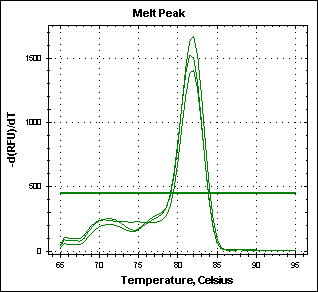 | 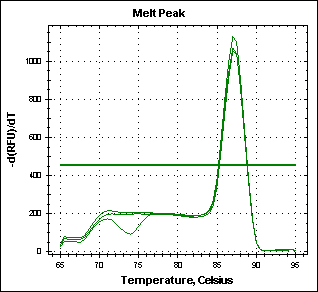 | 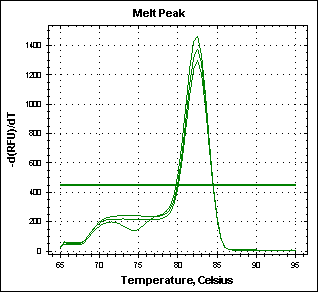 | 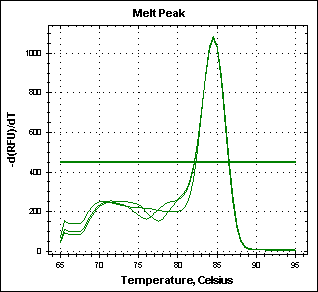 |
| RARA | SOCS3 | XBP1 |  |
| 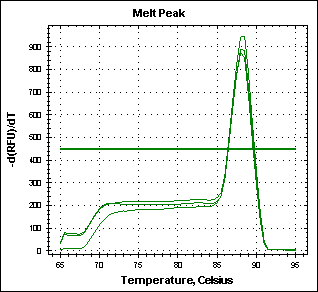 | 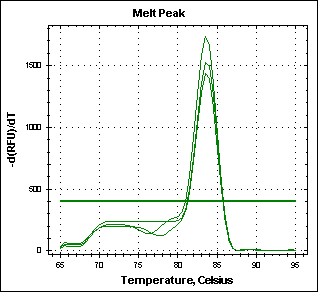 | 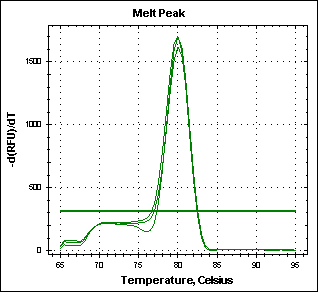 |  |

Supplementary Table 3: Representative melt-curve of qPCR products derived from RT-qPCR assay. Table present name of the gene, which mRNA expression was tested and graphic representation of meltcurve. Primers were derived from estrogen receptor signalling (SAB Target List) H384 Predesigned 384-well panel (Bio-Rad).

| l.p. | Method | Model | | | Minimal n | Nanoparticles | | |
| --- | --- | --- | --- | --- | --- | --- | --- | --- |
|  |  | MCF-7 | SK-BR-3 | *In vitro* |  | AgNPs | PSNPs | Mixture |
| 1 | NPs characterisation |  |  | X | 3 | X | X | X |
| 2 | NPs uptake assay | X | X |  | 3 | X | X | X |
| 3 | Viability | X | X |  | 6 | X | X |  |
| 4 | Proliferation | X |  |  | 6 | X | X | X |
| 5 | Wound healing assay | X | X |  | 12 | X | X | X |
| 6 | Cell cycle | X | X |  | 4 | X | X | X |
| 7 | ESR1 Polarisation assay |  |  | X | 20 | X | X |  |
| 8 | qPCR | X | X |  | 7 | X | X | X |

Supplementary Table 4: Overview of methods, models, minimal n and nanoparticles used in each experiment
